# Grand challenges for burrowing soft robots

**DOI:** 10.3389/frobt.2025.1525186

**Published:** 2025-02-13

**Authors:** Caitlin L. Le, Osman Dogan Yirmibesoglu, Sean Even, Trevor Buckner, Yasemin Ozkan-Aydin, Rebecca Kramer-Bottiglio

**Affiliations:** ^1^ Department of Mechanical Engineering and Materials Science, Yale University, New Haven, CT, United States; ^2^ Department of Electrical Engineering, University of Notre Dame, Notre Dame, IN, United States

**Keywords:** soft robotics, burrowing, bioinspiration, granular media, soil

## Abstract

Robotic burrowing holds promise for applications in agriculture, resource extraction, and infrastructure development, but current approaches are ineffective, inefficient, or cause significant environmental disruption. In contrast, natural burrowers penetrate substrates with minimal disturbance, providing biomechanical principles that could inspire more efficient and sustainable mechanisms. A notable feature of many natural burrowers is their reliance on soft body compositions, raising the question of whether softness contributes to their burrowing success. This review explores the role of soft materials in biological burrowing and their implications for robotic design. We examine the mechanisms that soft-bodied organisms and soft robots employ for submerging and subterranean locomotion, focusing on how softness enhances efficiency and adaptability in granular media. We analyze the gaps between the capabilities of natural burrowers and soft robotic burrowers, identify grand challenges, and propose opportunities to enhance robotic burrowing performance. By bridging biological principles with engineering innovation, this review aims to inform the development of next-generation burrowing robots capable of operating with the efficiency and efficacy seen in nature.

## 1 Introduction

Humans have long sought subterranean access for purposes such as agriculture, resource extraction, and infrastructure development (for example, tunnels for transit, underground electrical grids, or laying pipework). However, traditional human methods, including digging and media excavation, often result in substantial environmental disruption. In contrast, many natural burrowers are able to penetrate substrates without significantly altering the surrounding environment. This observation suggests that there are biomechanical principles to be gleaned from natural burrowers that could inform the development of more efficient, less invasive burrowing technologies (Che and Dorgan, 2010a; Dorgan, 2015).

A key feature of many natural burrowers is their partial or complete reliance on soft body compositions. This raises the question: Does the softness of their bodies contribute to their burrowing success? This review seeks to explore that question by examining the role of soft materials in biological burrowing and the lessons they offer for robotic design. Specifically, we review the literature on soft robots that mimic biological burrowing strategies, aiming to understand both the mechanisms of burrowing and the role of soft materials in robotic submerging and subterranean locomotion. A video format of this review with compiled videos and images of biological and robotic burrowing strategies can be viewed in Supplementary Video S1. This review complements other recent reviews on bioinspired planetary regolith-burrowing robots (Lopez-Arreguin and Montenegro, 2020; Wei et al., 2021), soft-bodied burrowing processes (Dorgan and Daltorio, 2023), mathematical modeling of burrowers (Hosoi and Goldman, 2015), and broader bioinspired approaches in geotechnical engineering (Martinez et al., 2021; Zhang W. et al., 2024).

### 1.1 Scope of this review

**FIGURE 1:**
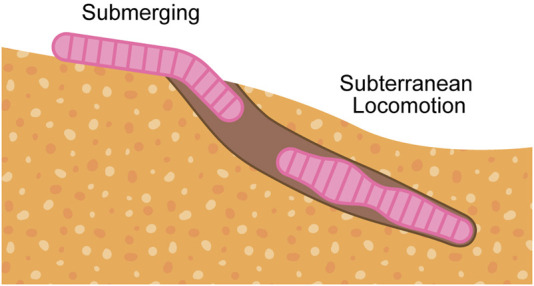
General flow of burrowing steps, which is composed of submerging (above-ground to below-ground) and subterranean locomotion (within-ground).

This review analyzes burrowing capabilities in soft-bodied organisms and soft robots, seeking to identify gaps in efficacy and propose opportunities to close the gaps. We limit our discussions to burrowers that are classified as soft through material properties or mechanical compliance. We also acknowledge excavating organisms (Jarvis and Sale, 1971; Suter et al., 2011) and rigid bioinspired robots (Kobayashi et al., 2011; Russell, 2011a; Lee et al., 2020; Treers et al., 2022; Zhang T. et al., 2024; Bagheri et al., 2024), incorporating them where their mechanisms overlap with those of soft-bodied burrowers. Our discussions on burrowing mechanisms are organized into two burrowing phases, illustrated in Figure 1: submerging, when going from above-ground to below-ground, and subterranean locomotion, when moving within the ground.

### 1.2 Variables that affect burrowing

Burrowing feasibility relies heavily on the physical properties of both the medium and the burrower. These properties dictate which mechanisms will be more or less effective, shaping the burrower’s ability to penetrate and navigate through specific media.

#### 1.2.1 Burrow media properties

Several factors affect the physical properties of burrow media, including grain size (diameter and shape of grains), moisture content (amount of water in the media), and cohesiveness (adhesion between grains) (Terzaghi et al., 1996). The factors combine in various ways to influence the behavior of the aggregate media. For burrowing, key aggregate properties include media failure and resistive forces.

The mechanical failure of burrow media is largely dependent on cohesiveness. Granular, cohesionless media tends to fail through radial collapse, or “landslide collapse,” where media begins to slide at a critical angle (Terzaghi et al., 1996; Lambe and Whitman, 1969). Radial collapse increases the forces required for burrowing, though Mohr’s circle analysis can help predict collapse (Isava and Winter 2016). In contrast, cohesive media can fail through fracture propagation, a process animals exploit to extend their burrows (Dorgan, 2015; Dorgan et al., 2005). Burrows in cohesive media tend to retain their shape, thus reducing resistive forces during burrowing. Some invertebrates, like earthworms and polychaetes, secrete mucus to media, temporarily increasing its cohesiveness for more efficient burrowing (Bottinelli et al., 2010; Pettibone, 1963).

In addition to the physical properties of burrow media and failure modes, resistive forces are largely affected by gravity since lithostatic pressure (the pressure the media exerts on a submerged body) increases linearly with depth (Terzaghi et al., 1996). This pressure gradient causes increasing drag during vertical burrowing (Robertson and Campanella, 1983) and lift during horizontal burrowing (Ding et al., 2011; Guillard et al., 2014).

#### 1.2.2 Burrower properties

For burrowing organisms, the leading tip shape affects the forces required for burrowing. Pointed tips, like shovel- or wedge-shaped heads, require lower forces than blunt faces (Maladen et al., 2011b). The sandfish skink, a type of lizard, features a shovel-shaped head for moving beneath sand, effectively reducing drag and lift forces (Maladen et al., 2009; Maladen et al., 2011b). In worms, wedge-shaped heads facilitate the expansion of cracks (Dorgan et al., 2005), aiding in locomotion within their environments.

Subterranean forces scale with body size. Larger bodies experience larger drag forces (Albert et al., 1999; Geng and Behringer, 2005; Wang et al., 2024) and, consequently, rely on specific burrowing mechanisms to counteract the bulk properties of their environments. For small burrowers, whose dimensions are comparable to or smaller than sediment grains, interactions with individual grains become more pronounced.

The surface properties of burrowers also influence resistive forces. Smooth skin reduces friction, as observed in sandfish skinks (Baumgartner et al., 2007). Some skin textures, like scales on snakes (Wu et al., 2020; Rieser et al., 2021) and setae on earthworms (Edwards and Bohlen, 1996), are anisotropic—producing high friction in one direction and low friction in another. This characteristic helps prevent unwanted backward movement.

**FIGURE 2:**
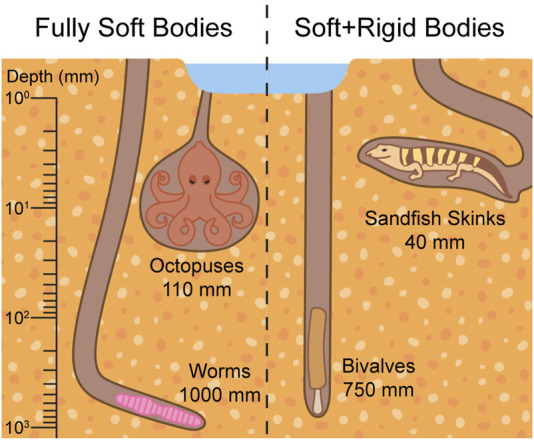
Burrowers with fully soft bodies and bodies made of both soft and rigid components reach similar depths. Worms can reach 1,000 mm (Cadman and Nelson-Smith, 1993), octopuses can reach 110 mm (Montana et al., 2015), bivalves can reach 750 mm (Holland and Dean, 1977), and sandfish skinks can reach 40 mm (Catena and Hembree, 2014).

The softness of burrowers’ bodies impacts the choice of burrowing mechanisms employed. For example, soft-bodied invertebrates rely on muscle contractions to change their body shape, like peristalsis in worms (Edwards and Bohlen, 1996). Snakes, which have both rigid and soft body parts, use their flexible body and ribs to scrape sand from around and beneath them (Young and Morain, 2003). Fully soft bodies, as well as combinations of soft and rigid body parts, enable successful burrowing at various depths, shown in Figure 2. We therefore infer that softness plays a critical role in shaping the burrowing strategies of organisms.

## 2 Soft biological burrowers

**FIGURE 3:**
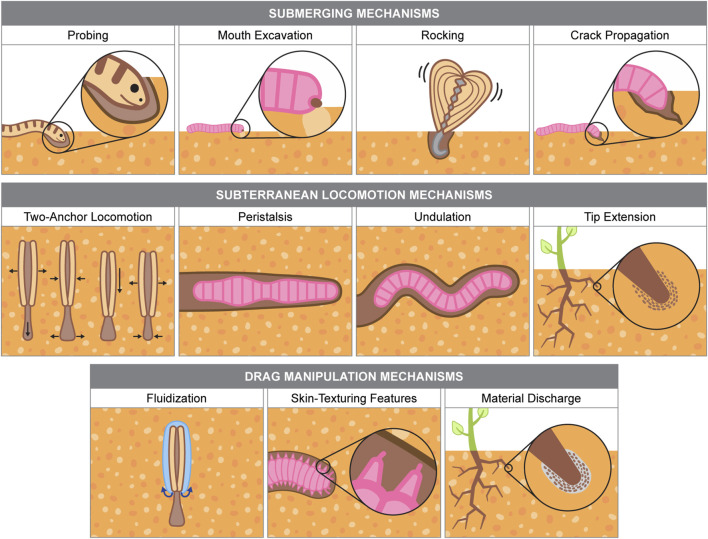
Burrowing mechanisms used by biological organisms. Information for burrowing organism examples are from the following sources: Sharpe et al. (2015), Trueman (1975), Trueman (1966), Trueman (1967), Hodge et al. (2009), Merz and Woodin (2006).

This section reviews flexible, soft-bodied organisms that burrow in granular substrates like dry sand and cohesive soil. Burrowing mechanisms are categorized by their roles in submerging and subterranean locomotion, with additional discussion on drag-modifying strategies. Some mechanisms, such as crack propagation and fluidization, support both submerging and subterranean movement. Illustrations and summaries of each mechanism appear in Figure 3; Tables 1, 2 outline burrower performance metrics.

**TABLE 1:** Burrowing worms. Width and length are body measurements perpendicular and parallel to the movement direction, respectively. Mean burrowing speed and maximum burrowing depth are reported in millimeters (mm) and body lengths (bl).

Organism	Approximate width (W), length (L)	Mean burrowing speed	Maximum burrowing depth	Burrow media	Submerging mechanism	Subterranean locomotion mechanism
Annelid worms (*Armandia brevis)* (Dorgan et al., 2013; Woodin, 1974)	W = 1 mm L = 20 mm	5 mm/s 0.25 bl/s	30 mm 1.5 bl	Wet sand, mud	Probing	Undulation
Cirratulid polychaetes (e.g., *Cirriformia moorei*, *Cirriformia grandis*) (Che and Dorgan, 2010a; Che and Dorgan, 2010b; Blake, 1996)	W = 1–6 mm L = 20–85 mm	0.02 mm/s 0.0004 bl/s	200 mm 3 bl	Wet sand, mud	Probing	Peristalsis, crack propagation
Earthworms (e.g., *Lumbricus terrestris*, *Nicodrilus giardi*) (Trueman, 1975; Edwards and Bohlen, 1996; James, 2022; Bastardie et al., 2005; Arrázola-Vásquez et al., 2022; Ruiz et al., 2017)	W = 2–10 mm L = 50–200 mm	0.1–0.5 mm/s 0.002 bl/s	600 mm 5 bl	Soil	Crack propagation, mouth excavation, probing	Crack propagation, mouth excavation, peristalsis, undulation
Glyceride polychaetes (Blood worms) (e.g., *Hemipodus simplex*, *Glycera dibranchiata*) (Murphy and Dorgan, 2011; Rizzo et al., 2007; Brown, 2015)	W = 2–4 mm L = 10–1,000 mm	0.06 mm/s 0.0001 bl/s	300 mm 0.6 bl	Wet sand, mud	Probing	Peristalsis, crack propagation
King ragworms (*Alitta virens*) (Dorgan et al., 2005; Pettibone, 1963; Dorgan et al., 2007; Dorgan et al., 2006; Hartman, 1969)	W = 45 mm L = 500–900 mm	8.7 mm/s 0.01 bl/s	450 mm 0.6 bl	Wet sand, mud	Crack propagation	Two-anchor locomotion
Lugworms (*Arenicola marina*, *Arenicola defodiens*) (Volkenborn et al., 2010; Marina, 1966; Riisgard and Banta, 1998; Cadman and Nelson-Smith, 1993)	W = 10 mm L = 200–400 mm	—	1,000 mm 3 bl	Wet sand	Probing, mouth excavation	Peristalsis, mouth excavation
Opheliid polychaetes (e.g., *Thoracophelia mucronata*) (Dorgan, 2018; Fox et al., 1948; Eikenberry, 1966)	W = 0.5–2 mm L = 30–50 mm	0.7 mm/s 0.02 bl/s	300 mm 8 bl	Wet sand	Probing	Peristalsis
Roundworms (*Caenorhabditis elegans*) (Lesanpezeshki et al., 2019; Beron et al., 2015; Palikaras and Tavernarakis, 2013)	W = 0.08 mm L = 1 mm	0.02 mm/s 0.02 bl/s (in agar)	—	Soil	—	Undulation

**TABLE 2:** Soft burrowing organisms. Width and length are body measurements perpendicular and parallel from movement direction, respectively. Length unit of body lengths (bl) is used in mean burrowing speed and maximum burrowing depth.

Organism	Approximate width (W), length (L)	Mean burrowing speed	Maximum burrowing depth	Burrow media	Submerging mechanism	Subterranean locomotion mechanism
Bivalve molluscs (e.g., *Mya arenaria*, *Cardium edule*) (Trueman, 1966; Checa and Cadée, 1997; Nel et al., 2001; Zwarts and Wanink, 1989)	W = 50 mm L = 80 mm	0.01 mm/s 0.0001 bl/s	180 mm 2 bl	Wet sand	Probing, rocking	Two-anchor locomotion, fluidization
Razor clams (bivalves) (e.g., *Ensis arcuatus*, *Ensis directus*) (Trueman, 1967; Winter et al., 2012; Jung et al., 2011; Holland and Dean, 1977)	W = 20 mm L = 100–300 mm	10 mm/s 0.05 bl/s	700 mm 3.5 bl	Wet sand	Probing, rocking	Two-anchor locomotion, fluidization
Burrowing eels (e.g., *Anguilla japonica*, *Anguilla anguilla*) (Aoyama et al., 2005; Steendam et al., 2020)	W = 300 mm L = 500–800 mm	—	300 mm 0.5 bl	Wet sand	Probing	Undulation
Plant roots (Hodge et al., 2009; Gilman, 1990)	W = 0–50 mm L = -	10–30 mm/day	30,000 mm	Soil, sand	Tip extension	Tip extension
Sand diving wrasses (e.g., *Halichoeres bivittatus*, *Cymolutes torquatus*) (Tatom-Naecker and Westneat, 2018)	W = 20 mm L = 90 mm	30 mm/s 0.3 bl/s	50 mm 0.6 bl	Wet sand	Probing	Undulation
Sand lances (e.g., *Ammodytes personatus*, *Ammodytes hexapterus*) (Gidmark et al., 2011; Bizzarro et al., 2016)	W = 5–10 mm L = 50–150 mm	100 mm/s 1 bl/s	150 mm 1.5 bl	Wet sand	Probing	Undulation
Sandfish skinks (lizard) (*Scincus scincus*, *Chalcides ocellatus*) (Maladen et al., 2009; Arnold, 1995; Catena and Hembree, 2014; Carranza et al., 2008)	W = 10 mm L = 100–135 mm	100 mm/s 0.9 bl/s	40 mm 0.3 bl	Dry sand	Probing	Undulation
Shovel-nosed snakes (e.g., *Chionactis occipitalis*) (Sharpe et al., 2015; Cowles, 1941; Klauber, 1951)	W = 10 mm L = 300–400 mm	50 mm/s 0.1 bl/s	600 mm 2 bl	Dry sand	Probing	Undulation
Snakeblennies (e.g., *Lumpenus lampretaeformis*) (Atkinson et al., 1987)	W = 15 mm L = 200 mm	0.01 mm/s 0.00005 bl/s	72 mm 0.36 bl	Wet sand	Probing	Undulation
Southern sand octopuses (*Octopus kaurna*) (Montana et al., 2015)	W = - L = 500 mm	10 mm/s 0.02 bl/s	110 mm 0.2 bl	Wet sand	Fluidization	Fluidization

### 2.1 Biological mechanisms for submerging

#### 2.1.1 Probing

Many soft-bodied animals use probing-based excavation as a mechanism to submerge and create an entry point or to penetrate existing cracks on the surface of subterranean environments. During probing, animals start at the surface of the substrate, with most of their body resting on it. The probing part—usually a pointed head or foot—extends towards the substrate and moves back and forth, manipulating the substrate to create an entry point large enough for the rest of the body to enter. The efficacy of this probing maneuver depends on the size, shape, and flexibility of the animal’s body, the type of burrow medium, and is also limited by the organism’s maximum muscle contraction rate. It is also important to note that the body must provide sufficient anchoring force for the insertion of a probe.

Probing-based submergence is common in animals with small body-diameter-to-length ratios that inhabit soft substrates. One example is the earthworm, which uses its prostomium, the anterior tip of the body, to create an entry point and a tunnel into the soil (Edwards and Bohlen, 1996). Similarly, shovel-nosed snakes probe their heads into a substrate to guide the rest of their bodies into a burrow (Sharpe et al., 2015). For bivalves, which are aquatic mollusks with hinged shells, the muscular foot extends from the head to create an entrance into sandy sea beds (Trueman, 1967). In these bivalves, the foot is a muscular outgrowth of the ventral body wall, which is used to propel the bivalve into the substrate. The foot first inserts into the granular environment and extends out from the shell. The subsequent anchoring and contraction of the foot pull the bivalve’s body forward, sliding it into the substrate.

#### 2.1.2 Mouth excavation

Through mouth excavation, an animal creates a burrow by ingesting the burrow media in front of it and expelling the digested material, or casts, behind it. Earthworms (Meysman et al., 2006) and lugworms (Volkenborn et al., 2010) commonly use mouth excavation for burrowing. This method is naturally slow, as it relies on an organism’s metabolic rate for digestion. Digestion can also alter the cohesiveness of the soil. For example, earthworms produce cohesive casts by adding water and intestinal mucus to the ingested soil, which helps them form a compact, stable path through the medium (Bottinelli et al., 2010).

#### 2.1.3 Rocking

Occurring in bivalves, this burrowing mechanism begins after the foot—a soft muscle that extends from the shell—penetrates the substrate. Contraction of body muscles causes extension of retractor muscles and changes the organism’s internal body pressure, producing a rocking motion of the shell (Trueman, 1966). During this rocking motion, rotation occurs about a fixed point between the body and the substrate surface. The body oscillates about this point, loosening the sediment at the burrow entrance, which reduces resistance and increases movement efficiency. Many bivalves also have complex sculptures on their shell surfaces, such as ridges, which are thought to aid the rocking motion by providing additional anchoring (Trueman, 1966; Stanley, 1969).

#### 2.1.4 Crack propagation

Crack propagation is an effective locomotion method that worms employ to enter and move through various soils with minimal resistance and energy expenditure (Dorgan et al., 2005; Dorgan et al., 2007). To propagate cracks forward, worms evert their pharynx to apply excessive loading on the granular medium and create a fracture. The forces required for crack propagation, as well as the effectiveness of crack locomotion, depend on many factors, including the medium properties [softer soils are easier to move through than harder soils (Ruiz et al., 2015)], crack depth [shallower cracks offer less resistance than deeper cracks (Terzaghi et al., 1996)], and tip size [a smaller tip requires less energy to propagate cracks than a larger tip (Li et al., 2013)].

### 2.2 Biological mechanisms for subterranean locomotion

#### 2.2.1 Two-anchor locomotion

Some animal burrowers, such as worms and clams, form anchors to apply forward forces without resurfacing or moving backward (Trueman, 1975; Dorgan, 2015). In the burrowing process, these animals alternate advancing the anterior (front) and posterior (back) parts of their body, with penetration and terminal anchoring formed sequentially as they burrow downward (Trueman, 1967). For example, razor clams radially expand the posterior end of their body, or their shell, to create a penetration anchor that prevents backward movement, allowing them to extend and generate downward thrust at the anterior tip of their body, or their foot. Then, the anterior tip radially expands to create the terminal anchor, facilitating the retraction of the posterior part of the body to advance forward. To create these bulbous anchors, body fluid is transferred between the anterior and posterior ends (Trueman, 1967). Other organisms that exhibit the two-anchor locomotion behavior may also burrow with similarly-shaped parts, like their head or proboscis, a tubular appendage that extends from the mouth (Dorgan, 2015).

#### 2.2.2 Peristalsis

In peristalsis, waves of muscle contractions move forward (anterograde) or backward (retrograde) along a body. Like two-anchor locomotion, penetration and terminal anchors propagate throughout the body to generate continuous and directed motion. *Polyphysia*, a burrower with a continuous hydrostatic skeleton, features circular and longitudinal muscles arranged along the organism’s body; contraction of one type of muscle forces incompressible coelomic, or body, fluid to displace along the length of the body, creating propagations that propel the organism forward (Elder, 1973). In septate soft-bodied animals, like earthworms, the volume of individual body segments is constant and the segments extend only when their diameter is reduced (Edwards and Bohlen, 1996). The alternating pattern of peristalsis involves the contraction of the circular muscles to extend the segment, followed by the contraction of the longitudinal muscles to shorten and increase the diameter of the segment which anchors the body to the medium, resulting in forward movement. Depending on the type of body cavity, continuous or segmented, the anchoring points travel along the body in the posterior or anterior direction of the animal (Trueman, 1975; Seymour, 1971).

#### 2.2.3 Undulation

Burrowing by undulation is used by several organisms that feature flexible, elongated bodies (Maladen et al., 2009; Dorgan et al., 2013; Gray and Lissmann, 1964; Gidmark et al., 2011; Ozkan-Aydin et al., 2021a; Herrel et al., 2011). With rapid undulation of their bodies, organisms like lizards (Maladen et al., 2009; Sharpe et al., 2015), snakes (Sharpe et al., 2015), worms (Ozkan-Aydin et al., 2021a), and fish (Atkinson et al., 1987), can push against and relocate environmental media around their bodies to generate propulsion. Burrowing with undulation is possible in cohesionless and cohesive media, like dry sand or muddy ground (Maladen et al., 2009; Dorgan et al., 2013; Atkinson and Pullin, 1996). Animals typically match their undulation amplitude, wavelength, and frequency to the intended granular material (Rieser et al., 2024; Pierce et al., 2024).

To shallowly cover their bodies with granular media, flatfish (McKee et al., 2016) and sand vipers (Young and Morain, 2003) also use body undulations to flick sand over themselves. Flatfish undulate their bodies at a high frequency to quickly kick up sand, while Saharan sand vipers undulate sections of their bodies to cover themselves slowly, starting with covering their posterior end and moving to their head. These organisms use undulations for submerging purposes, rather than for subterranean locomotion purposes.

#### 2.2.4 Tip extension

In plant roots, tip growth occurs through cell division and expansion at the apex. Cells in the meristem divide to form new cells, which elongate behind the meristem to lengthen the root (Hodge et al., 2009). These new cells, initially small, elongate rapidly while mature cells remain stationary. This focused growth minimizes soil penetration resistance, as only the small, growing tip interacts with the soil (Hodge et al., 2009). Additionally, some roots grow in a helical manner, or circumnutation behavior, which helps roots increase penetration distance in soil (Fisher, 1964; Taylor et al., 2021). This inherent “gait” allows roots to continually grow in different directions to avoid becoming stuck when encountering obstacles, and is also thought to reduce the penetration resistance in granular media (Del Dottore et al., 2016).

### 2.3 Auxiliary mechanisms for drag manipulation

Depending on their size and burrowing depth, organisms have specialized adaptations to reduce drag while burrowing and increase drag while anchoring. We previously discussed the effects of body shape on intrusion forces and drag, such as a pointed probe for insertion into granular media and expanding body segments for anchoring. In addition to body shape, organisms have other mechanisms and features to alter the drag on their bodies, including fluidization, skin-texturing, and material discharge.

#### 2.3.1 Fluidization

By ejecting water into surrounding granular media, organisms can reduce the forces needed to burrow vertically. Many bivalves (Trueman et al., 1966) and Pacific sandfish (MacDonald, 2015), found in wet granular environments, extract water from their surroundings and eject it in the direction of intended burrowing. Local fluidization suspends granular particles in fluid, allowing the body to quickly advance downward before the particles settle and reconsolidate (Winter et al., 2013). In the two-anchor locomotion process of bivalves, fluidization occurs when the foot extends further and when the shell is pulled down. To lower frictional forces around the foot, water is ejected only around the foot, rather than the shell that needs to anchor. Similarly, when pulling the shell down, water ejection is around the mantle, located in the posterior end, to fluidize only around the posterior body part that needs to move, rather than the foot that needs to anchor (Trueman et al., 1966). Octopuses also eject water into the seafloor, using fluidization as the primary method to submerge themselves (Montana et al., 2015). Sandfish lizards undulate their bodies with large amplitudes, creating a localized fluid-like zone around their bodies (Maladen et al., 2009).

#### 2.3.2 Skin-texturing features

Many undulating and oscillating animals have smaller features on their skin that vary frictional forces depending on the direction of motion. On snake skin, scales have texture (Wu et al., 2020; Rieser et al., 2021) and can be controlled to increase friction with the environment (Marvi et al., 2016). Scales have microscopic steps that have directionality, resulting in a higher-friction skin when measuring from tail-to-head compared to from head-to-tail (Wu et al., 2020). This anisotropic friction is beneficial for gripping the environment when undulating forward, but is not direction-dependent when sidewinding (Rieser et al., 2021). Snakes also actuate their scales, opening and extending them from their skin, doubling the friction forces (Marvi et al., 2016). Like the microscopic features on snake scales, chaetae on polychaetes (Merz and Woodin, 2006) and setae on earthworms (Edwards and Bohlen, 1996) also provide anisotropic friction. Both of these rigid, bristle-like structures prevent backward movement during locomotion, providing higher friction for anchoring.

#### 2.3.3 Material discharge

Friction reduction between plant roots and soil is achieved by sloughing, or shedding, cells at the root cap (Bengough and Mckenzie, 1997) and secretion of mucilage, a gelatinous substance (Guinel and McCully, 1986). When growing, new cells added at root tips may become attached to the root or detached. Detached cells act as a reduced-friction liner between the root and soil (Bengough and Mckenzie, 1997). In addition to cell sloughing, root caps secrete mucilage to lubricate between root caps and soil. The friction reduction by mucilage is heavily determined by the hydration of the mucilage, and it is thought that the role of mucilage as a lubricant is relatively minor (Guinel and McCully, 1986).

## 3 Soft robotic burrowers

**FIGURE 4:**
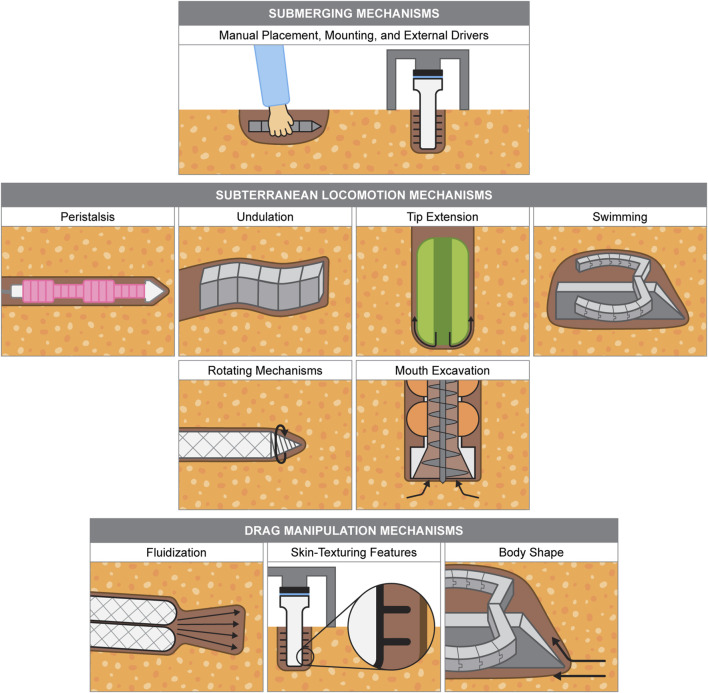
Burrowing mechanisms used by soft robots. Example robot drawings are based from the following: Das et al. (2020), Sadeghi et al. (2013), Eken et al. (2023), Niiyama et al. (2022), Maladen et al. (2011a), Isaka et al. (2019), Tang et al. (2024), Chopra et al. (2023), Naclerio et al. (2021).

Biological burrowing systems exhibit a remarkable diversity of strategies and adaptations, providing a valuable source of inspiration for designing advanced burrowing technologies. By exploring the unique mechanisms these organisms use to navigate and interact with their environments, we gain critical insights to inform the development of efficient, adaptable, and resilient robotic burrowing systems. Like biological examples, burrowing robots range from being composed of entirely soft materials, combinations of soft and rigid materials, and mechanisms that afford flexible behavior. Here, we present the established mechanisms that have been successfully demonstrated in robotic prototypes, mirroring the order of the mechanisms demonstrated by the biological examples presented in Section 2 where possible. Some robotic burrowing mechanisms listed are not found in biology, like rotating mechanisms, while others have not yet been achieved by robots, like crack propagation. We also include examples of mechanisms that could be leveraged for burrowing or subterranean locomotion, although not implemented on a burrowing robot. Burrowing mechanism illustrations are shown in Figure 4, and a summary table of soft burrowing robots is given in Table 3.

**TABLE 3:** Soft burrowing robots organized by bioinspiration. Width and length are body measurements perpendicular and parallel to the movement direction, respectively. Burrowing speed is in the horizontal (horiz.) or vertical (vert.) direction, being perpendicular or parallel to the direction of gravity, respectively. Length unit of body lengths (bl) is used in maximum burrowing speed and maximum burrowing depth.

Robot	Width (W), length (L)	Maximum burrowing speed	Maximum burrowing depth	Material	Burrow media	Submerging mechanism	Subterranean locomotion mechanism	Bioinspiration
Flagella robot (Du et al., 2022)	W = 20 mm L = 140 mm	0.6 mm/s 0.004 bl/s (horiz.)	—	Plastic, silicone	Water beads in a tube	Manual placement	Rotating mechanism	Bacteria
Vine robot (Naclerio et al., 2021)	W = 60 mm L = 2,000 mm (fully everted)	4,800 mm/s (horiz.)	350 mm	Ripstop nylon	Dry sand	Mount	Tip extension, fluidization	Plant roots
Untethered vine robot (Eken et al., 2023)	W = 16 mm L = 320 mm	3.92 mm/s 0.01 bl/s (vert.)	40 mm 0.1 bl	Thermoplastic polyurethane	Glass beads	Manual placement	Tip extension	Plant roots
Root-inspired robot (Sadeghi et al., 2013)	W = 20 mm L = 40 mm	1 mm/s 0.03 bl/s (vert.)	40 mm 1 bl	Textile fabric	Glass beads	Mount	Tip extension	Plant roots
RootBot (Han et al., 2024)	W = 200 mm L = -	0.01 mm (horiz.)	0 mm (exposed to surface)	Silicone	Sand	Mount	Tip extension, rotating mechanism, excavation	Plant roots
Rotating, horizontal-burrowing robot (Tang et al., 2024)	W = 18 mm L = 211.6 mm	0.08 mm/s 0.0004 bl/s (horiz.)	80 mm 0.4 bl	Silicone	Dry glass beads	Manual placement	Rotating mechanism, extension-contraction	Razor clams, seeds, lizards
Snake-inspired robot (Even et al., 2023)	D = 55 mm L = 640 mm	3 mm/s 0.005 bl/s (vert.)	170 mm 0.3 bl	Plastic, linked servomotors	Packing peanuts	Undulation	Undulation	Saharan sand vipers
Sand-swimming robot (Maladen et al., 2011a)	W = 54 mm L = 480 mm	144 mm/cycle 0.3 bl/cycle (horiz.)	4 mm 0.008 bl	Linked servomotors	Plastic beads	Manual placement	Undulation	Sandfish skinks
Anisotropic-appendaged swimmer (Chopra et al., 2023)	W = 51 mm L = 256 mm	1.6 mm/s 0.006 bl/s (horiz.)	127 mm 0.5 bl	Plastic	Sand	Manual placement	Swimming	Sea turtles, sandfish skinks
Bristle-worm-inspired robot (Ortiz et al., 2019)	W = 25 mm L = 210 mm	0.16 mm/s 0.0008 bl/s (horiz.)	80 mm 0.4 bl	Silicone	Polypropylene pellets	Manual placement	Two-anchor locomotion	Bristle worms
Bristle-worm-inspired robot (Drotman et al., 2022)	W = 40 mm L = 40 mm	0.2 mm/s 0.005 bl/s (horiz.)	40 mm 1 bl	Plastic	Dry sand	Manual placement	Two-anchor locomotion	Bristle worms
Peristaltic crawling robot (Omori et al., 2009)	W = 60 mm L = 300 mm	17 mm/s 0.06 bl/s (vert.)	1,000 mm 3 bl	Plastic	Acrylic tubes, dirt tunnels	Manual placement	Peristalsis	Earthworms
Earthworm-inspired robot (Das et al., 2020)	W = 35 mm L = 350 mm	4.38 mm/s 0.01 bl/s (horiz.)	25 mm 0.07 bl	Silicone, braided sleeve	Plastic beads	Manual placement	Peristalsis	Earthworms
Earthworm-inspired robot (Niiyama et al., 2022)	W = 10 mm L = 132 mm	0.025 mm/s 0.0002 bl/s (horiz.)	5 mm 0.04 bl	Rubber-like resin	Soil	Manual placement	Peristalsis	Earthworms
SEAVO II (Isaka et al., 2019)	W = 150 mm L = 650 mm	6.8 mm/s 0.01 bl/s (vert.)	430 mm 0.7 bl	Aluminum, rubber	Underwater soil	Mount	Peristalsis, rotating mechanism, excavation	Earthworms

### 3.1 Soft robot mechanisms for submerging

Most soft robots require assistance from an external hand, machine, or mount to achieve successful submersion. Here, we highlight the various submerging mechanisms employed by soft robots in the literature, which naturally overlap with the biological mechanisms described in Section 2.1.

#### 3.1.1 Manual placement, mounting, and external drivers

A substantial number of soft burrowing robots have no submersion mechanism, instead being placed *in situ* by reporting authors (Ortiz et al., 2019; Tao et al., 2020; Chopra et al., 2023; Maladen et al., 2011a; Eken et al., 2023; Tang et al., 2024; Du et al., 2022; Omori et al., 2009; Das et al., 2020; Niiyama et al., 2022; Drotman et al., 2022). Similarly, some robots are deployed off of mounts, rods, or launchers held above granular media beds (Naclerio et al., 2021; Sadeghi et al., 2017; Sadeghi et al., 2013; Isaka et al., 2019; Han et al., 2024), showing that their submerging methods are feasible but lack control of their submersion direction.

If a robot cannot generate sufficient propulsive force to submerge itself, an external machine can be used to drive it into the granular medium. In such cases, the robot’s design focuses on steering, reducing drag, or loosening soil, while the external system supplies the submersion force. This approach is ideal for shallow burrowing, testing friction mitigation methods, or performing auxiliary functions. Submersion can be achieved using a robotic arm to push the robot (Naclerio et al., 2021) or a tether to pull it downward (Germann et al., 2011), often with force gauges or other sensors to assess effectiveness. Similar techniques are employed in endoscopic medical devices, which navigate small tunnels without disturbing walls (Fagogenis et al., 2019), making this method useful when depth is secondary and a tethered connection is acceptable.

#### 3.1.2 Probing

In practice, probing has been achieved with the assistance of a mount or launching mechanism that holds the robot above a surface. Two notable probing robots are rigid burrowers designed with specialized geometries to clear a path for the body to follow (Sadeghi et al., 2017; Winter et al., 2014). A root-inspired robot employs a parabolic head (Sadeghi et al., 2017), while a razor-clam-inspired robot features a hemispherical foot (Winter et al., 2014). Probing mechanisms are actuated through various methods, including tip growth via 3D printing (Sadeghi et al., 2017), extension with a piston (Winter et al., 2014), and hammering (Olaf et al., 2019). Notably, there is a lack of soft probing robots, likely due to the challenges of controlling soft continuum bodies, as observed in earthworms and bivalves (discussed in Section 2.1.1), and a primary focus on testing subterranean locomotion methods.

### 3.2 Soft robot mechanisms for subterranean locomotion

Once a burrowing soft robot is below the surface, the next step is to exhibit some form of subterranean locomotion. Very few soft robots are capable of driving through a granular medium, so we also include related demonstrations, including locomotion through enclosed tunnels.

#### 3.2.1 Two-anchor locomotion

Taking inspiration from live clams and their ability to self-anchor, the two-anchor mechanism has been studied extensively (see Section 2.2.1) and implemented in several robotic adaptations. A worm-inspired robot pneumatically expands and contracts the front and back of its body, similar to a razor clam (Ortiz et al., 2019). Another soft robot has passively-actuated anisotropic structures located on the front and back sections of the robot (Drotman et al., 2022). By lengthening and shortening the robot’s body, two-anchor locomotion is achieved with the setae-inspired structures. The two-anchor strategy has also been used in other non-burrowing worm-inspired robots, commonly pneumatically actuated, for locomotion in pipes or preexisting tubular burrows, using suction cups (Zhang et al., 2021), skin-textured and high-friction expanding anchors (Jiang and Pei, 2021; Liu et al., 2019; Zarrouk et al., 2012), and plain expanding anchors (Connolly et al., 2015; Calderon et al., 2016; Liu et al., 2022; Zhang et al., 2019).

Other razor-clam-inspired robots use methods resembling two-anchor locomotion. To return to the surface, one soft robot uses a pneumatically-actuated silicone tube wrapped with strain-limiting threads to elongate and shorten its body, relying on the pressure gradient of the granular environment to lift the robot upwards (Tao et al., 2020; Tao et al., 2019). A rigid razor-clam-inspired robot features only a single terminal anchor actuated by a pneumatic piston, which quickly actuates to take advantage of the fluidized granular environment (Winter et al., 2014).

#### 3.2.2 Peristalsis

Peristalsis, inspired by earthworms, is another approach to subterranean locomotion. Soft robots typically consist of two or more radially and axially expanding pneumatic components connected in series, enabling them to grip tunnel walls (Das et al., 2020; Isaka et al., 2019). Alternatively, motors (Omori et al., 2009) or shape memory alloy actuators (Niiyama et al., 2022) can be used to expand and contract segmented chambers. By activating these actuators in a wave-like pattern, the robots propel themselves forward, pulling their trailing ends behind.

#### 3.2.3 Undulation

Like peristalsis, undulating robots are created in segments to move in a wave-like manner, either with rigid links (Maladen et al., 2011a) or flexible chambers (Qi et al., 2020; Ozkan-Aydin et al., 2021b). A sandfish-skink-inspired robot undulates with seven rigid segments actuated by motors, with a wedge-shaped head to prevent resurfacing during horizontal locomotion (Maladen et al., 2011c). Pneumatic segments comprise tube-traveling worm-inspired robots that can bend and turn within tube networks (Qi et al., 2020; Ozkan-Aydin et al., 2021b). Although these robots are not demonstrated to burrow, the undulation mechanisms have the potential to be successful in creating underground tunnels.

Undulation is another method for submerging, focusing on lifting and moving soil to create pockets for movement, rather than forcing the robot into the soil. This approach was studied in a robotic flatfish model, where higher undulation frequencies enhanced burrowing ability (McKee et al., 2016). The undulating silicone model generated turbulence, displacing loose sand to clear space around the body. Similarly, a snake-inspired robot mimics the burrowing behavior of Saharan sand vipers, undulating to move granular media and sink into it (Even et al., 2023). While sand vipers use undulation to cover their bodies, the robotic version burrows deeper into the environment.

#### 3.2.4 Tip extension

Tip extension, inspired by plant root growth, is a strategy closely related to head probing. In this case, rather than forcing an intruder into the soil, the intruder tip grows into the soil while the remainder of the robot is fixed. Since the bulk of the robot remains stationary, the vast majority of drag forces are eliminated.

“Vine robots,” a popular class of tip extension robots, involve everting an internal sleeve of material into the burrow media (Hawkes et al., 2017; Naclerio et al., 2021; Sadeghi et al., 2013; Coad et al., 2019). This is typically achieved by inflating an inverted plastic sheath from a stationary base holding the power sources and spool of material. The burrow path can be passively controlled by pre-forming the sleeve or actively controlled with tendons pulling at the tip (Naclerio et al., 2021; Coad et al., 2019). Vine robots can also feature burrowing tools, such as rotating cutterheads to clear the burrow ahead (Han et al., 2024). Traditionally, these robots are limited to a predefined distance based on the spool length, though an untethered version has shown promise for burrowing independently, using motorized rollers to evert a toroidal plastic body (Eken et al., 2023).

In another example, a robot grows by 3D printing its body along the way. A small 3D-printing head rides on the robot’s tip and continuously extrudes a shell behind it, as long as filament is fed through the robot body (Sadeghi et al., 2017; Del Dottore et al., 2024). To control the direction of the robot, the printing head can be tilted, depositing material unevenly and turning the front tip of the robot. Similar to vine robots, the travel distance of printing robots is limited to the amount of filament they have access to.

Also inspired from plant roots, circumnutation has been utilized and studied in robotic systems, lowering required penetration energy in granular media (Del Dottore et al., 2016; Del Dottore et al., 2017) and increasing locomotion success of worm-inspired robots in obstacle-filled environments (Ozkan-Aydin et al., 2019; Even and Ozkan-Aydin, 2023).

#### 3.2.5 Swimming

Swimming robots scoop and pull granular media behind them, similar to swimming breaststroke in water (Russell, 2011b; Chopra et al., 2021; Li et al., 2021). Robots typically use asymmetrically flexible appendages, such that they are rigid during the power portion of the stroke, propelling the robot forward, and can easily be brought forward through the soil with lower drag during the recovery portion of the stroke. In practice, these anisotropic appendages have been constructed using a single paddle (Russell, 2011b), a chain of rigid links (Chopra et al., 2021), or a combination of segments attached to a continuous sheet (Li et al., 2021), all designed to straighten and propel the robot forward while passively bending during the recovery stroke.

#### 3.2.6 Rotating mechanisms

Moving beyond bioinspiration, robots can take advantage of infinitely-rotating joints, which do not exist in biological organisms. One robot uses a soft, pneumatically driven tube that extends and contracts with a rotating cone at its tip (Tang et al., 2024). Some robots feature links of auger segments, which allow the robot body to bend while retaining its drilling capability (Nagai et al., 2017; Fujiwara et al., 2018). Aside from drilling mechanisms, bacteria-inspired robots have been shown to continuously rotate flagella-like limbs to locomote through water beads (Du et al., 2022).

#### 3.2.7 Mouth excavation

Like biological burrowers, some robotic burrowers also excavate media out of their burrows. One vine robot scrapes sand with dual rotating cutterheads, sprays water into the sand, then suctions the material out of the tunnel (Han et al., 2024). This vine robot has many components located on an off-board base, including the pump used for vacuuming. Another excavating example is an earthworm-inspired robot with a soft, pneumatic outer skin around a rigid internal auger, which takes in burrow media through the front and expels it from the rear when burrowing downward in underwater soil (Isaka et al., 2019). Three segments of outer chambers take turns expanding and moving forward, mimicking peristaltic motion to move the robot down while drilling. As the drilled soil can simply be relocated behind the robot, there is a reduced need to push and compact the soil against the tunnel walls to open a gap.

### 3.3 Auxiliary mechanisms for drag manipulation

Burrowing robots can be designed with mechanisms or features to manipulate frictional forces between the robot and granular media. In some cases, increased friction is useful for anchoring the main robot body, while reduced friction cuts down on drag. Below, we discuss drag manipulation approaches utilized in recent robotics literature.

#### 3.3.1 Fluidization

As with burrowing organisms, fluidization involves suspending solid particles in a fluid to form a fluid-like material that reduces drag forces against the surface of the robot during burrowing. One approach to achieving fluidization is by injecting fluid into granular media, as demonstrated by a vine-inspired robot that jets air into dry sand (Naclerio et al., 2021). This study also showed that higher fluidization rates correlate with reduced drag. However, jetting fluid may not be feasible for all robots, as it requires large pumps or fluid reservoirs. In contrast, a razor-clam-inspired robot anchors itself into the environment by expanding its body and then rapidly contracts to create a surrounding volume of fluidized media, thereby lowering the forces required for burrowing (Winter et al., 2014). Additionally, mechanical vibrations have been shown to reduce penetration forces in granular media, though they have not yet been implemented in robotic platforms (Darbois Texier et al., 2017).

#### 3.3.2 Skin-texturing features

As discussed in Section 2.3.2, the small hair-like structures on animal skin create surfaces with varying friction, which are advantageous for both anchoring in granular media and facilitating sliding for locomotion. A root-inspired vine robot has small spikes of thermal glue on its textile skin to help the everting body grip the environment (Sadeghi et al., 2013). An earthworm-inspired pneumatic robot has sheaths of kirigami skins on both ends of its body, which actuate when the ends inflate for anchoring and retract when the ends deflate for locomotion (Liu et al., 2019). Another worm-inspired pneumatic robot has passive setae-inspired features that are anisotropic depending on movement direction, acting as anchors for two-anchor locomotion (Drotman et al., 2022). The setae sit against the robot body when moving forward in a low-drag state, but when the robot is pushed back, the setae open up to prevent backward movement. Rigid aluminum plates are also used as setae-inspired features on an earthworm-inspired robot (Isaka et al., 2019). Similarly, another earthworm-inspired robot has static bristle-like features that have a wedge shape to allow easier movement in the forward direction and higher drag in the backward direction (Niiyama et al., 2022). On a larger scale, origami appendages on a reciprocating burrowing robot also provide anisotropic forces during subterranean locomotion (Kim et al., 2023).

#### 3.3.3 Body shape

The geometry of the head, fins, or other appendages on a burrowing robot plays a significant role in its ability to move through soil or other granular materials. By designing these features with specific shapes, friction can be minimized, enhancing the robot’s burrowing performance in both vertical and horizontal locomotion. For vertical penetration, less blunt intrusion tips reduce the forces required for burrowing (Zaidi and Müller, 2017; Ye and Zhang, 2023). For horizontal locomotion, wedge-shaped heads (Naclerio et al., 2021; Maladen et al., 2011c) reduce drag and lift forces (Ding et al., 2011). As robots move forward in the horizontal plane, an angled head assists in pushing the robot downward to avoid resurfacing. Angled control surfaces have also been added to wedge-shaped heads to increase surface area and counteract net lift forces acting on the robot (Chopra et al., 2023; Drotman et al., 2022).

## 4 Grand challenges

**FIGURE 5:**
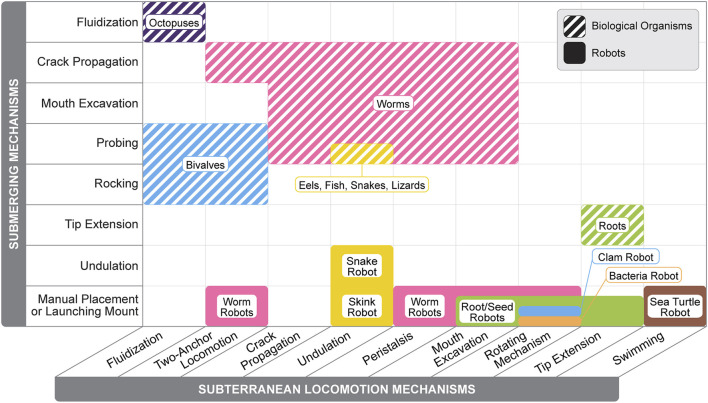
Comparison between burrowing methods employed by soft biological organisms and robots. Burrowing methods are divided into submerging mechanisms (vertical axis) and subterranean locomotion mechanisms (horizontal axis). Information for burrowing methods are from the following sources: Dorgan et al. (2013), Che and Dorgan (2010a), Trueman (1975), Murphy and Dorgan (2011), Dorgan et al. (2005), Volkenborn et al. (2010), Dorgan (2018), Lesanpezeshki et al. (2019), Trueman (1966), Trueman (1967), Aoyama et al. (2005), Hodge et al. (2009), Tatom-Naecker and Westneat (2018), Gidmark et al. (2011), Maladen et al. (2009), Sharpe et al. (2015), Atkinson et al. (1987), Montana et al. (2015), Du et al. (2022), Naclerio et al. (2021), Eken et al. (2023), Sadeghi et al. (2013), Han et al. (2024), Tang et al. (2024), Even et al. (2023), Maladen et al. (2011a), Chopra et al. (2023), Ortiz et al. (2019), Omori et al. (2009), Das et al. (2020), Niiyama et al. (2022), Isaka et al. (2019).

**FIGURE 6:**
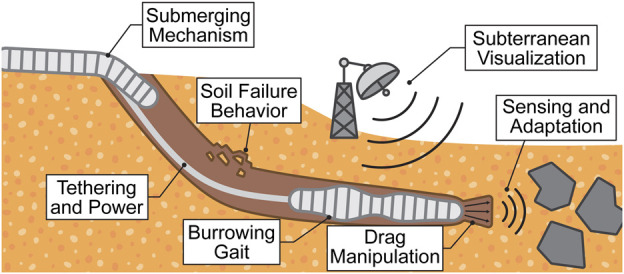
Summary of the discussed grand challenges soft burrowing robots face in granular media. In this illustration, the robots are in an environment with cohesionless granular media, but the represented challenges apply for cohesive media as well.

It is evident that robots do not match the burrowing performance of natural organisms. Unlike their biological counterparts, robots often fail to employ effective submerging methods and face other limitations, as illustrated in Figure 5. Due to their larger size and heavier components, robots experience significantly greater forces during burrowing and locomotion. This increased scale and mass amplify resistance from surrounding substrates, requiring more force to penetrate, displace, or move through dense media. Consequently, robots exhibit lower gait efficiency, typically quantified by the Cost of Transport (CoT) metric: CoT=P/mgv , where P is power consumed, m is the robot’s mass, g is gravitational acceleration, and v is the robot’s velocity. In this section, we highlight and examine the grand challenges in developing efficient, soft burrowing robots, as illustrated in Figure 6.

### 4.1 Effective submerging mechanisms

We must address the challenge of developing autonomous submerging mechanisms for soft robots to eliminate the need for manual placement or external intervention, as discussed in Section 3.1.1. While manual placement or launching mounts may be sufficient for some applications, those requiring above-surface locomotion or full autonomy must address the submersion problem.

Many soft burrowing robots, inspired by organisms that use probing to submerge, feature conical or tip-like geometries optimized for penetration (see Section 3.3.3). However, these robots often lack the ability to orient themselves from a horizontal above-ground position to an angled or vertical position necessary for ground penetration. For vine robots, directional control methods used underground—such as preformed paths or tendon-driven guidance—can also be applied to direct the robot downward into the substrate (Naclerio et al., 2021; Coad et al., 2019). Similarly, soft worm- and bivalve-inspired robots can utilize multi-chamber pneumatic structures, commonly employed in undulating robots, to articulate and aim their soft bodies (see Section 3.2.3). Robots with rigid bodies, which are unable to penetrate the ground directly (Chopra et al., 2021), could incorporate deployable launching arms designed specifically for submersion tasks. For instance, a linkage arm that deploys from beneath the robot to angle it downward could function similarly to the deployable landing legs used on reusable rocket boosters (SpaceX, 2021).

Additionally, soft robotic systems can draw inspiration from biological submersion methods, as illustrated in Figure 5. One promising approach, crack propagation, has been theoretically explored but not yet implemented on physical robots (Lathrop and Paley, 2021). The primary challenge lies in creating soft, crack-expanding mechanisms that are small enough to enter soil cracks or identifying cracks large enough to accommodate existing mechanisms. For soft worm-inspired robots, the disparity between their size and the tiny micro-cracks present in soil poses significant barriers to effective submersion (Das et al., 2023; Liu J. et al., 2023). Developing small, robust soft penetration mechanisms would mark a significant advancement in self-submerging soft burrowing robotics, bridging the size mismatch between robots and natural soil cracks.

### 4.2 Drag manipulation strategies

Burrowing robots must manage the balance between high drag for anchoring and low drag for efficient forward movement in granular media. While robots have successfully employed bioinspired friction manipulation techniques (see Section 3.3.2), there is still room for improvement to enhance burrowing efficiency. The following sections explore various methods for friction manipulation to address this challenge.

#### 4.2.1 High-friction and anchoring mechanisms

Robust anchoring mechanisms are essential for soft robots to prevent backward motion during submerging and locomotion gaits in granular media. For untethered systems or those without access to environmental fluids, robots can utilize enclosed body fluids to grow body sections for anchoring, similar to the mechanism used by razor clams (Trueman, 1967). Traditional pumps can facilitate fluid relocation, but an alternative approach involves fully soft microfluidic systems that use chemical reactions to control flow (Wehner et al., 2016). For soft ballooning anchors, reinforcement is crucial to prevent rupturing under high pressures, a risk that increases at greater depths due to the need for higher internal pressure to maintain performance (Shepherd et al., 2013). Additionally, puncture resistance is vital in granular media containing sharp edges or when interacting with rigid robot components. Soft anchoring mechanisms must be designed to resist punctures and tears while maintaining sufficient stiffness to withstand high external pressures at depth without failure (Martinez et al., 2014). Materials with these properties are key, and incorporating strain-limiting threads can provide structural support to soft anchors.

Anisotropic skin features used by robots often take the form of large appendages or scales rather than fine skin textures. Their size necessitates significant movement and deformation to fully splay out and anchor, leading to larger backward displacements and reduced net-forward progress. Moreover, large gaps between these features and the robot body can become filled with granular media, diminishing their anisotropic effectiveness. A promising alternative is the use of smaller skin features, similar in scale to fingerprints or gecko spatulae (Autumn and Peattie, 2002), which require less actuation for effective anisotropic behavior. However, these features must be larger than the voids in the granular media to ensure effective anchoring. High-friction materials can also enhance anchoring but may hinder movement during the gait cycle if used on parts that require mobility. Shape-changing surfaces present another innovative solution, enabling spiked features to splay out as the surface curves (Sun et al., 2020; Wang and Chortos, 2024). Such surfaces are particularly effective for anchoring, as inflating a body segment with splayed spikes can significantly reduce backward movement and improve overall performance.

#### 4.2.2 Drag reduction and fluidization mechanisms

Mitigating drag forces is a critical challenge for improving the efficiency and reducing the energy consumption of soft burrowing robots in granular media. Fluidization through jetting fluid is a proven technique for reducing drag (see Section 3.3.1), but it typically requires a tether to external pumps and reservoirs. Developing a tetherless design poses significant challenges, including the need for compact, high-powered pumps and a mechanism to extract and store fluid from the environment for jetting purposes.

An alternative to fluid jetting is vibration, which necessitates a rigid interface between the robot and its environment since a soft body naturally dampens vibrations. Implementing vibration in a soft burrowing robot would require a method for dynamically adjusting the robot’s stiffness, such as pneumatically actuated variable stiffness systems (Baines et al., 2023; Fitzgerald et al., 2020). Additionally, robots can exploit environmental conditions and their own natural movements to induce a fluid-like state in the soil, similar to the locomotion strategies of sandfish lizards (Maladen et al., 2009). Exploring fluidization methods that do not rely on fluid jetting could reduce tethering requirements and lower power demands, paving the way for smaller and more efficient burrowing robots.

Finally, the environmental impact of fluidization techniques must be carefully considered, particularly in fragile ecosystems. Sustainable and eco-friendly fluidization methods are essential to minimize ecological disruption and ensure compatibility with diverse environments.

### 4.3 Burrowing gait optimization

Burrowing robots, like biological systems, must adaptively optimize their gaits to the depth and material properties of the substrates they encounter. Such adaptability is exemplified by razor clams, which increase the period of their two-anchor locomotion at greater depths to achieve deeper penetration (Trueman, 1967), and by plant roots, which use circumnutation behavior to navigate obstacles and reduce burrowing energy expenditure. Key features of burrowing gaits, such as period, frequency, and characteristic motions, must dynamically adjust to the soil conditions.

Simulations using resistive force theory and the discrete element method offer drag force approximations that inform planned burrowing gaits based on granular media properties (Hosoi and Goldman, 2015). However, the underground environment is inherently unpredictable, with conditions constantly shifting. This unpredictability highlights the potential of data-driven techniques to outperform predefined gaits. Recent advances in machine learning demonstrate the ability to dynamically optimize burrowing strategies in complex, variable environments. For instance, a study on a snake-like burrowing robot employed a novel deep-learning architecture to refine self-burrowing strategies, outperforming traditional methods and successfully navigating granular media (Even et al., 2023). These findings underscore the promise of data-driven approaches in enabling robots to autonomously adapt their gaits to environmental changes, thereby enhancing their subterranean performance in real time.

### 4.4 Sensor development and integration

Burrowing robots must integrate real-time sensing to navigate the challenges of dynamic underground environments, characterized by variable soil properties and limited visibility. In the absence of visual cues, inertial measurement units (IMUs) can track position, orientation, and motion relative to gravity, though they often struggle with noise-induced position errors. Tactile sensors are also critical for detecting soil compaction changes or obstacles, such as rocks or roots, by measuring environmental forces on the robot’s limbs, enabling adjustments to avoid becoming stuck (Yuan et al., 2017). Additionally, soil moisture measurement is key for predicting soil behavior. A recent study employed resistivity, humidity, and optical reflectance sensors to monitor moisture levels, informing real-time adjustments to burrowing strategies and improving efficiency and resilience (Del Dottore et al., 2023).

While advancements in soft, flexible, and stretchable sensors show promise, most available sensors remain rigid, potentially limiting flexibility in soft robot designs (Liu E. et al., 2023). Recent progress in hybrid and stretchable circuits offers solutions for integrating sensors into soft robots (Valentine et al., 2017; Liu et al., 2021; Woodman et al., 2024). However, sensors also increase power demands, presenting an additional challenge for energy-constrained systems.

### 4.5 Untethering soft burrowing robots

A significant challenge for burrowing robots, and robots in general, is transitioning from tethered systems to fully autonomous operation while maintaining reliable power and performance in underground environments. Tethers offer advantages in high-stakes scenarios, such as search-and-rescue missions, where they provide a dependable power source and enable remote control (Murphy, 2004). However, for applications like environmental or habitat monitoring, tethers can be disruptive, potentially damaging ecosystems or scaring away animals. Additionally, tethers introduce drawbacks such as increased drag and load as the robot penetrates deeper into granular media (Ambaye et al., 2024; Su et al., 2022), risks of entanglement, and reduced maneuverability. These limitations hinder the ability of tethered soft burrowing robots to effectively navigate complex underground environments.

The development of tetherless, autonomous soft burrowing robots is essential to overcoming these constraints. One approach involves integrating onboard power and locomotion systems, such as pneumatic mechanisms. However, existing pumping technologies face a tradeoff between size and power output. Similarly, for battery-powered solutions, there is a tradeoff between battery size, operational duration, and drag. Larger batteries extend operational time but also increase the robot’s load and drag, complicating movement through dense media.

Energy harvesting technologies are also being explored to enhance operational efficiency and reduce reliance on stored energy. Piezoelectric systems, for example, generate electricity from mechanical pressure (Lu et al., 2023), which could be leveraged as burrowing robots encounter increasing pressure with depth (Terzaghi et al., 1996). Thermoelectric generators, which convert temperature gradients into electricity (Yang et al., 2021), could similarly harness environmental energy in areas with temperature variation. These innovations hold promise for enabling robots to supplement their energy needs directly from their surroundings.

### 4.6 Other considerations

Roboticists face numerous challenges, including advancements in batteries, actuators, soft materials, and sensor fusion (Du et al., 2022). For burrowing robots, two additional challenges stand out: granular media failure and subterranean visualization.

Preventing granular media collapse is essential for smoother and more efficient burrowing. One unexplored approach is adding liquid to the environment to create cohesive media that can be compacted, mimicking the strategy used by earthworms. Another method is bracing the burrow walls, as demonstrated by vine robots that grow via tip extension (Coad et al., 2019). Exploring alternative bracing strategies beyond tip growth could further enhance stability and efficiency.

Subterranean visualization of a robot is critical for understanding and predicting burrowing robot behavior. Conventional techniques, such as ground-penetrating radar (Wickramanayake et al., 2022), x-ray imaging (Maladen et al., 2011), and acoustic tracking (Zhang et al., 2022), have provided valuable insights into the interactions between robots and granular materials. For instance, one study employed magnetic field strength as a proxy for depth in a snake-inspired robot, offering a practical and efficient solution (Even et al., 2023). While these methods are highly effective in controlled laboratory settings, they face significant challenges, including high costs, complex integration with robotic systems, and substantial power demands. Additionally, the size and weight of the required equipment often make them impractical for real-world applications, where portability and ease of deployment are important.

## 5 Conclusion

This review highlights the diverse mechanisms employed by biological organisms to effectively submerge and navigate within granular media. From small insects to larger burrowers, animals demonstrate efficient strategies across various scales and environments, offering valuable inspiration for robotic burrowing. Current bioinspired robotic systems have made strides in mimicking these biological movements but often lag in efficiency, largely due to the challenges of miniaturizing robots with the necessary power and dexterity. Specifically, limitations in submersion techniques and difficulties in maintaining effective locomotion within dense substrates restrict the practicality of soft robotic burrowers.

To address these gaps, we recommend standardized metrics for evaluating burrowing robots, such as maximum burrowing speed and energy costs (e.g., CoT ), as well as measurements specific to vertical and horizontal locomotion depths. Drawing on the partial or complete reliance on soft body compositions in natural burrowers, we hypothesize that softness plays a critical role in enabling more efficient and effective burrowing. For instance, soft-bodied burrowers may be better equipped to navigate complex or shifting substrates. However, validating this hypothesis requires a direct comparison between rigid and soft robots. Developing standardized evaluation metrics will be essential to assess the performance of soft versus rigid robotic burrowers under comparable conditions and to quantify the true benefits of softness in burrowing efficiency.

Closing the gap between natural and robotic burrowers will support the development of efficient, minimally invasive robots for environmental and extraterrestrial exploration. We hope this review encourages advancements that bring soft burrowing robotics closer to the adaptability and efficiency displayed by natural burrowers.

## References

[B1] Albert R. Pfeifer M. A. Barabási A.-L. Schiffer P. (1999). Slow drag in a granular medium. Phys. Rev. Lett. 82, 205–208. 10.1103/PhysRevLett.82.205

[B2] Ambaye G. Boldsaikhan E. Krishnan K. (2024). Soft robot design, manufacturing, and operation challenges: a review. J. Manuf. Mater. Process. 8, 79. 10.3390/jmmp8020079

[B3] Aoyama J. Shinoda A. Sasai S. Miller M. J. Tsukamoto K. (2005). First observations of the burrows of Anguilla japonica. J. Fish Biol. 67, 1534–1543. 10.1111/j.1095-8649.2005.00860.x

[B4] Arnold E. N. (1995). Identifying the effects of history on adaptation: origins of different sand-diving techniques in lizards. Tech. Rep. 235, 351–388. 10.1111/j.1469-7998.1995.tb01758.x

[B5] Arrázola-Vásquez E. Larsbo M. Capowiez Y. Taylor A. Sandin M. Iseskog D. (2022). Earthworm burrowing modes and rates depend on earthworm species and soil mechanical resistance. Appl. Soil Ecol. 178, 104568. 10.1016/j.apsoil.2022.104568

[B6] Atkinson R. J. Pelster B. Bridges C. R. Taylor A. C. Morris S. (1987). Behavioural and physiological adaptations to a burrowing lifestyle in the snake blenny, Lumpenus lampretaeformis, and the red band-fish, Cepola rubescens. J. Fish Biol. 31, 639–659. 10.1111/j.1095-8649.1987.tb05268.x

[B7] Atkinson R. J. A. Pullin R. S. V. (1996). Observations on the burrows and burrowing behaviour of the red band-fish, cepola rubescens l. Mar. Ecol. 17, 23–40. 10.1111/j.1439-0485.1996.tb00487.x

[B8] Autumn K. Peattie A. M. (2002). Mechanisms of adhesion in geckos. Integr. Comp. Biol. 42, 1081–1090. 10.1093/icb/42.6.1081 21680391

[B9] Bagheri H. Stockwell D. Bethke B. Okwae N. K. Aukes D. Tao J. (2024). A bio-inspired helically driven self-burrowing robot. Acta Geotech. 19, 1435–1448. 10.1007/s11440-023-01882-9

[B10] Baines R. Yang B. Ramirez L. A. Kramer-Bottiglio R. (2023). Kirigami layer jamming. Extreme Mech. Lett. 64, 102084. 10.1016/j.eml.2023.102084

[B11] Bastardie F. Capowiez Y. Cluzeau D. (2005). 3d characterisation of earthworm burrow systems in natural soil cores collected from a 12-year-old pasture. Appl. Soil Ecol. 30, 34–46. 10.1016/j.apsoil.2005.01.001

[B12] Baumgartner W. Saxe F. Weth A. Hajas D. Sigumonrong D. Emmerlich J. (2007). The sandfish’s skin: morphology, chemistry and reconstruction. J. Bionic Eng. 4, 1–9. 10.1016/S1672-6529(07)60006-7

[B13] Bengough A. G. Mckenzie B. M. (1997). Sloughing of root cap cells decreases the frictional resistance to maize (Zea mays L.) root growth. Tech. Rep. 309. 10.1093/jxb/48.4.885

[B14] Beron C. Vidal-Gadea A. G. Cohn J. Parikh A. Hwang G. Pierce-Shimomura J. T. (2015). The burrowing behavior of the nematode caenorhabditis elegans: a new assay for the study of neuromuscular disorders. Genes, Brain Behav. 14, 357–368. 10.1111/gbb.12217 25868909 PMC4444045

[B15] Bizzarro J. J. Peterson A. N. Blaine J. M. Balaban J. P. Greene H. G. Summers A. P. (2016). Burrowing behavior, habitat, and functional morphology of the pacific sand lance (Ammodytes personatus). Fish. Bull. 114, 445–460. 10.7755/FB.114.4.7

[B16] Blake J. A. (1996). Family cirratulidae ryckholdt, 1851 including a revision of the genera and species from the eastern north pacific. Taxonomic Atlas Benthic Fauna Santa Maria Basin West. Santa Barbara Channel 6, 263–384.

[B17] Bottinelli N. Hallaire V. Menasseri-Aubry S. Le Guillou C. Cluzeau D. (2010). Abundance and stability of belowground earthworm casts influenced by tillage intensity and depth. Soil Tillage Res. 106, 263–267. 10.1016/j.still.2009.11.005

[B18] Brown B. (2015). Maine’s baitworm fisheries: resources at risk? Am. Zoologist 33, 568–577. 10.1093/icb/33.6.568

[B19] Cadman P. S. Nelson-Smith A. (1993). A new species of lugworm: arenicola defodiens sp. nov. J. Mar. Biol. Assoc. U. K. 73, 213–223. 10.1017/s0025315400032744

[B20] Calderon A. A. Ugalde J. C. Zagal J. C. Perez-Arancibia N. O. (2016). Design, fabrication and control of a multi-material-multi-actuator soft robot inspired by burrowing worms. IEEE Int. Conf. Robotics Biomimetics, 31–38doi. 10.1109/ROBIO.2016.7866293

[B21] Carranza S. Arnold E. N. Geniez P. Roca J. Mateo J. (2008). Radiation, multiple dispersal and parallelism in the skinks, chalcides and sphenops (squamata: scincidae), with comments on scincus and scincopus and the age of the sahara desert. Mol. phylogenetics Evol. 46, 1071–1094. 10.1016/j.ympev.2007.11.018 18276164

[B22] Catena A. M. Hembree D. I. (2014). Swimming through the substrate: the neoichnology of chalcides ocellatus and biogenic structures of sand-swimming vertebrates. Palaeontol. Electron. 17. 10.26879/463

[B23] Che J. Dorgan K. M. (2010a). It’s tough to be small: dependence of burrowing kinematics on body size. J. Exp. Biol. 213, 1241–1250. 10.1242/JEB.038661 20348335

[B24] Che J. Dorgan K. M. (2010b). Mechanics and kinematics of backward burrowing by the polychaete Cirriformia moorei. J. Exp. Biol. 213, 4272–4277. 10.1242/jeb.049320 21113009

[B25] Checa A. G. Cadée G. C. (1997). Hydraulic burrowing in the bivalve *Mya arenaria* linnaeus (Myoidea) and associated ligamental adaptations. J. Molluscan Stud. 63, 157–171. 10.1093/mollus/63.2.157

[B26] Chopra S. Jadhav S. Tolley T. M. Gravish N. (2021). Mechanical and actuation asymmetry in soft appendages leads to robotic propulsion in granular media

[B27] Chopra S. Vasile D. Jadhav S. Tolley M. T. Gravish N. (2023). Toward robotic sensing and swimming in granular environments using underactuated appendages. Adv. Intell. Syst. 5, 2200404. 10.1002/aisy.202200404

[B28] Coad M. M. Blumenschein L. H. Cutler S. Zepeda J. A. R. Naclerio N. D. El-Hussieny H. (2019). Vine robots. IEEE Robotics and Automation Mag. 27, 120–132. 10.1109/mra.2019.2947538

[B29] Connolly F. Polygerinos P. Walsh C. J. Bertoldi K. (2015). Mechanical programming of soft actuators by varying fiber angle. Soft Robot. 2, 26–32. 10.1089/soro.2015.0001

[B30] Cowles R. B. (1941). Observations on the winter activities of desert reptiles. Ecology 22, 125–140. 10.2307/1932207

[B31] Darbois Texier B. Ibarra A. Melo F. (2017). Low-resistive vibratory penetration in granular media. PloS one 12, e0175412. 10.1371/journal.pone.0175412 28419123 PMC5395245

[B32] Das R. Babu S. P. M. Palagi S. Mazzolai B. (2020). “Soft robotic locomotion by peristaltic waves in granular media,” in 2020 3rd IEEE international conference on soft robotics (RoboSoft) (IEEE), 223–228.

[B33] Das R. Babu S. P. M. Visentin F. Palagi S. Mazzolai B. (2023). An earthworm-like modular soft robot for locomotion in multi-terrain environments. Sci. Rep. 13, 1571. 10.1038/s41598-023-28873-w 36709355 PMC9884293

[B34] [Dataset] Hodge A. Berta G. Doussan C. Merchan F. Crespi M. (2009). Plant root growth, architecture and function. Plant Soil 321, 153–187. 10.1007/s11104-009-9929-9

[B35] Del Dottore E. Mondini A. Bray D. Mazzolai B. (2023). “Miniature soil moisture sensors for a root-inspired burrowing growing robot,” in Conference on biomimetic and biohybrid systems (Springer), 184–196.

[B36] Del Dottore E. Mondini A. Rowe N. Mazzolai B. (2024). A growing soft robot with climbing plant–inspired adaptive behaviors for navigation in unstructured environments. Sci. Robotics 9, eadi5908. 10.1126/scirobotics.adi5908 38232147

[B37] Del Dottore E. Mondini A. Sadeghi A. Mattoli V. Mazzolai B. (2016). “Circumnutations as a penetration strategy in a plant-root-inspired robot,” in 2016 IEEE international conference on robotics and automation (ICRA) (IEEE), 4722–4728.

[B38] Del Dottore E. Mondini A. Sadeghi A. Mattoli V. Mazzolai B. (2017). An efficient soil penetration strategy for explorative robots inspired by plant root circumnutation movements. Bioinspiration and biomimetics 13, 015003. 10.1088/1748-3190/aa9998 29123076

[B39] Ding Y. Gravish N. Goldman D. I. (2011). Drag induced lift in granular media. Phys. Rev. Lett. 106, 028001. 10.1103/physrevlett.106.028001 21405251

[B40] Dorgan K. Jumars P. Johnson B. Boudreau B. (2006). Macrofaunal burrowing. 85–122. 10.1201/9781420006391.ch3

[B41] Dorgan K. M. (2015). The biomechanics of burrowing and boring. J. Exp. Biol. 218, 176–183. 10.1242/JEB.086983 25609781

[B42] Dorgan K. M. (2018). Kinematics of burrowing by peristalsis in granular sands. J. Exp. Biol. 221, jeb167759. 10.1242/jeb.167759 29636410

[B43] Dorgan K. M. Arwade S. R. Jumars P. A. (2007). Burrowing in marine muds by crack propagation: kinematics and forces. J. Exp. Biol. 210, 4198–4212. 10.1242/jeb.010371 18025018

[B44] Dorgan K. M. Daltorio K. A. (2023). Fundamentals of burrowing in soft animals and robots. Front. Robot. AI 10, 1057876. 10.3389/frobt.2023.1057876 36793873 PMC9923007

[B45] Dorgan K. M. Jumars P. A. Johnson B. Boudreau B. P. Landis E. (2005). Burrow extension by crack propagation. Nature 433, 475. 10.1038/433475a 15690029

[B46] Dorgan K. M. Law C. J. Rouse G. W. (2013). Meandering worms: mechanics of undulatory burrowing in muds. Proc. R. Soc. B Biol. Sci. 280, 20122948. 10.1098/rspb.2012.2948 PMC3619478 23446526

[B47] Drotman D. Chopra S. Gravish N. Tolley M. T. (2022). “Anisotropic forces for a worm-inspired digging robot,” in 2022 IEEE 5th international conference on soft robotics (RoboSoft), 261–266. 10.1109/RoboSoft54090

[B48] Du Y. Lam J. Sachanandani K. Jawed M. K. (2022). Modeling the locomotion of articulated soft robots in granular medium. IEEE Robotics Automation Lett. 7, 6495–6502. 10.1109/lra.2022.3173036

[B49] Edwards C. Bohlen P. (1996). “Biology and ecology of earthworms,” in Biology and ecology of earthworms (Netherlands: Springer).

[B50] Eikenberry A. B. (1966). A study of the vertical and horizontal migrations of euzonus (thoracophelia) mucronata (treadwell) 1914, on pacific coast beaches with regard to environmental factors

[B51] Eken K. Gravish N. Tolley M. T. (2023). “Continuous skin eversion enables an untethered soft robot to burrow in granular media,” in 2023 IEEE international conference on soft robotics (RoboSoft) (IEEE), 1–6.

[B52] Elder H. Y. (1973). Direct peristaltic progression and the functional significance of the dermal connective tissues during burrowing in the polychaete polyphysia crassa (oersted). J. Exp. Biol. 58, 637–655. 10.1242/jeb.58.3.637

[B53] Even S. Gordon H. Yang H. Ozkan-Aydin Y. (2023). Machine learning-driven burrowing with a snake-like robot

[B54] Even S. Ozkan-Aydin Y. (2023). “Locomotion and obstacle avoidance of a worm-like soft robot,” in 2023 IEEE/RSJ international conference on intelligent robots and systems (IROS) (IEEE).

[B55] Fagogenis G. Mencattelli M. Machaidze Z. Rosa B. Price K. Wu F. (2019). Autonomous robotic intracardiac catheter navigation using haptic vision. Sci. Robotics 4, eaaw1977. 10.1126/scirobotics.aaw1977 PMC6693882 31414071

[B56] Fisher J. E. (1964). Evidence of circumnutational growth movements of rhizomes of poa pratensis l. that aid in soil penetration. Can. J. Bot. 42, 293–299. 10.1139/b64-024

[B57] Fitzgerald S. G. Delaney G. W. Howard D. (2020). A review of jamming actuation in soft robotics. Actuators 9, 104. 10.3390/act9040104

[B58] Fox D. L. Crane S. C. McConnaughey B. H. (1948). A biochemical study of the marine annelid worm, thoracophelia mucronata: its food, biochromes and carotenoid metabolism

[B59] Fujiwara A. Nakatake T. Tadami N. Isaka K. Yamada Y. Sawada H. (2018). “Development of both-ends supported flexible auger for lunar earthworm-type excavation robot leavo,” in 2018 IEEE/ASME international conference on advanced intelligent mechatronics (AIM), 924–929. 10.1109/AIM.2018.8452676

[B60] Geng J. Behringer R. P. (2005). Slow drag in two-dimensional granular media. Phys. Rev. E 71, 011302. 10.1103/PhysRevE.71.011302 15697590

[B61] Germann D. P. Schatz W. Hotz P. E. (2011). Artificial bivalves - the biomimetics of underwater burrowing. Procedia Comput. Sci. 7, 169–172. 10.1016/j.procs.2011.09.012

[B62] Gidmark N. J. Strother J. A. Horton J. M. Summers A. P. Brainerd E. L. (2011). Locomotory transition from water to sand and its effects on undulatory kinematics in sand lances (Ammodytidae). J. Exp. Biol. 214, 657–664. 10.1242/jeb.047068 21270315

[B63] Gilman E. F. (1990). Tree root growth and development. i. form, spread, depth and periodicity. J. Environ. Hortic. 8, 215–220. 10.24266/0738-2898-8.4.215

[B64] Gray J. Lissmann H. W. (1964). The locomotion of nematodes. J. Exp. Biol. 41, 135–154. 10.1242/jeb.41.1.135 14161604

[B65] Guillard F. Forterre Y. Pouliquen O. (2014). Lift forces in granular media. Phys. Fluids 26, 043301. 10.1063/1.4869859

[B66] Guinel F. McCully M. (1986). Some water-related physical properties of maize root-cap mucilage. Plant, Cell and Environ. 9, 657–666. 10.1111/j.1365-3040.1986.tb01624.x

[B67] Han G. Seo D. Ryu J.-H. Kwon T.-H. (2024). Rootbot: root-inspired soft-growing robot for high-curvature directional excavation. Acta Geotech. 19, 1365–1377. 10.1007/s11440-023-02073-2

[B68] Hartman O. (1969). *Atlas of the errantiate polychaetous Annelids from California* allan hancock foundation. Los Angeles: University of Southern California.

[B69] Hawkes E. W. Blumenschein L. H. Greer J. D. Okamura A. M. (2017). A soft robot that navigates its environment through growth. Sci. Robotics 2, eaan3028–8. 10.1126/scirobotics.aan3028 33157883

[B70] Herrel A. Choi H. F. Dumont E. De Schepper N. Vanhooydonck B. Aerts P. (2011). Burrowing and subsurface locomotion in anguilliform fish: behavioral specializations and mechanical constraints. J. Exp. Biol. 214, 1379–1385. 10.1242/jeb.051185 21430215

[B71] Holland A. Dean J. (1977). The biology of the stout razor clamtagelus plebeius: I. animal-sediment relationships, feeding mechanism, and community biology. Chesap. Sci. 18, 58–66. 10.2307/1350364

[B72] Hosoi A. E. Goldman D. I. (2015). Beneath our feet: strategies for locomotion in granular media, Annu. Rev. Fluid Mech., 47, 431–453. 10.1146/annurev-fluid-010313-141324

[B73] Isaka K. Tsumura K. Watanabe T. Toyama W. Sugesawa M. Yamada Y. (2019). Development of underwater drilling robot based on earthworm locomotion. Ieee Access 7, 103127–103141. 10.1109/access.2019.2930994

[B74] Isava M. Winter A. G. (2016). Razor clam-inspired burrowing in dry soil. Int. J. Non-Linear Mech. 81, 30–39. 10.1016/j.ijnonlinmec.2015.12.005

[B75] James S. (2022). Lumbricus terrestris. CABI Compendium. 10.1079/cabicompendium.109385

[B76] Jarvis J. U. Sale J. B. (1971). Burrowing and burrow patterns of east african mole-rats tachyoryctes, heliophobius and heterocephalus. J. Zoology 163, 451–479. 10.1111/j.1469-7998.1971.tb04544.x

[B77] Jiang C. Pei Z. (2021). An in-pipe worm robot with pneumatic actuators based on origami paper-fabric composites. Text. Res. J. 91, 2724–2737. 10.1177/00405175211016561

[B78] Jung S. Winter A. G. Hosoi A. E. (2011). Dynamics of digging in wet soil. Int. J. Non-Linear Mech. 46, 602–606. 10.1016/j.ijnonlinmec.2010.11.007

[B79] Kim S. Treers L. K. Huh T. M. Stuart H. S. (2023). Efficient reciprocating burrowing with anisotropic origami feet. Front. Robotics AI 10, 1214160. 10.3389/frobt.2023.1214160 PMC10433778 37600474

[B80] Klauber L. M. (1951). The shovel-nosed snake, chionactis, with descriptions of two new subspecies , 11, 141, 204. 10.5962/bhl.part.28860

[B81] Kobayashi T. Tshukagoshi H. Honda S. Kitagawa A. (2011). “Burrowing rescue robot referring to a mole’s shoveling motion,” in Proceedings of the 8th JFPS international symposium on fluid power, 644–649.

[B82] Lambe T. W. Whitman R. V. (1969). Soil mechanics. Wiley.

[B83] Lathrop J. P. Paley D. A. (2021). Burrowing locomotion via crack propagation of a bio-inspired soft robot. IFAC-PapersOnLine 54, 128–133. 10.1016/j.ifacol.2021.11.164

[B84] Lee J. Tirtawardhana C. Myung H. (2020). “Development and analysis of digging and soil removing mechanisms for mole-bot: bio-inspired mole-like drilling robot,” in 2020 IEEE/RSJ international conference on intelligent robots and systems (IROS) (IEEE), 7792–7799. 10.1109/IROS45743.2020.9341230

[B85] Lesanpezeshki L. Hewitt J. E. Laranjeiro R. Antebi A. Driscoll M. Szewczyk N. J. (2019). Pluronic gel-based burrowing assay for rapid assessment of neuromuscular health in c. elegans. Sci. Rep. 9, 15246. 10.1038/s41598-019-51608-9 31645584 PMC6811592

[B86] Li C. Zhang T. Goldman D. I. (2013). A terradynamics of legged locomotion on granular media. Science 339, 1408–1412. 10.1126/science.1229163 23520106

[B87] Li D. Huang S. Tang Y. Marvi H. Tao J. Aukes D. M. (2021). Compliant fins for locomotion in granular media. IEEE Robotics Automation Lett. 6, 5984–5991. 10.1109/lra.2021.3084877

[B88] Liu B. Ozkan-Aydin Y. Goldman D. I. Hammond F. L. (2019). “Kirigami skin improves soft earthworm robot anchoring and locomotion under cohesive soil,” in RoboSoft 2019 - 2019 IEEE international conference on soft robotics (April: Institute of Electrical and Electronics Engineers Inc.), 828–833. 10.1109/ROBOSOFT.2019.8722821

[B89] Liu E. Cai Z. Ye Y. Zhou M. Liao H. Yi Y. (2023a). An overview of flexible sensors: development, application, and challenges. Sensors 23, 817. 10.3390/s23020817 36679612 PMC9863693

[B90] Liu J. Li P. Zuo S. (2023b). Actuation and design innovations in earthworm-inspired soft robots: a review. Front. Bioeng. Biotechnol. 11, 1088105. 10.3389/fbioe.2023.1088105 36896011 PMC9989016

[B91] Liu S. Shah D. S. Kramer-Bottiglio R. (2021). Highly stretchable multilayer electronic circuits using biphasic gallium-indium. Nat. Mater. 20, 851–858. 10.1038/s41563-021-00921-8 33603186

[B92] Liu X. Song M. Fang Y. Zhao Y. Cao C. (2022). Worm-inspired soft robots enable adaptable pipeline and tunnel inspection. Adv. Intell. Syst. 4, 2100128. 10.1002/aisy.202100128

[B93] Lopez-Arreguin A. Montenegro S. (2020). Towards bio-inspired robots for underground and surface exploration in planetary environments: an overview and novel developments inspired in sand-swimmers. Heliyon 6, e04148. 10.1016/j.heliyon.2020.e04148 32613101 PMC7317692

[B94] Lu J. Miao Z. Wang Z. Liu Y. Zhu D. Yin J. (2023). Piezoelectric soft robot driven by mechanical energy. Nano Res. 16, 4970–4979. 10.1007/s12274-022-5180-y

[B95] MacDonald I. (2015). *Burial mechanics of the Pacific sandfish: The role of the ventilatory pump and physical constraints on the behavior*. Master’s thesis. Ann Arbor: Northern Arizona University, ProQuest Dissertations Publishing.

[B96] Maladen R. Ding Y. Umbanhowar P. Kamor A. Goldman D. I. (2011). Biophysically inspired development of a sand-swimming robot. 10.7551/mitpress/9123.001.0001

[B97] Maladen R. D. Ding Y. Li C. Goldman D. I. (2009). Undulatory swimming in sand: subsurface locomotion of the sandfish lizard. Science 325, 314–318. 10.1126/science.1172490 19608917

[B98] Maladen R. D. Ding Y. Umbanhowar P. B. Goldman D. I. (2011a). Undulatory swimming in sand: experimental and simulation studies of a robotic sandfish. Int. J. Robotics Res. 30, 793–805. 10.1177/0278364911402406

[B99] Maladen R. D. Umbanhowar P. B. Ding Y. Masse A. Goldman D. I. (2011b). “Granular lift forces predict vertical motion of a sand-swimming robot,” in *2011 IEEE international Conference on Robotics and automation* (IEEE), 1398–1403.

[B100] Maladen R. D. Umbanhowar P. B. Ding Y. Masse A. Goldman D. I. (2011c). “Granular lift forces predict vertical motion of a sand-swimming robot,” in Proceedings - IEEE international conference on robotics and automation, 1398–1403. 10.1109/ICRA.2011.5980301

[B101] Marina A. L. (1966). Observations on the burrowing of arenicola marina (L.), 93–118. 10.1242/jeb.44.1.93 5922741

[B102] Martinez A. DeJong J. Akin I. Aleali A. Arson C. Atkinson J. (2021). Bio-inspired geotechnical engineering: principles, current work, opportunities and challenges. 72, 687, 705. 10.1680/jgeot.20.P.170

[B103] Martinez R. V. Glavan A. C. Keplinger C. Oyetibo A. I. Whitesides G. M. (2014). Soft actuators and robots that are resistant to mechanical damage. Adv. Funct. Mater. 24, 3003–3010. 10.1002/adfm.201303676

[B104] Marvi H. Cook J. P. Streator J. L. Hu D. L. (2016). Snakes move their scales to increase friction. Biotribology 5, 52–60. 10.1016/j.biotri.2015.11.001

[B105] McKee A. MacDonald I. Farina S. C. Summers A. P. (2016). Undulation frequency affects burial performance in living and model flatfishes. Zoology 119, 75–80. 10.1016/j.zool.2015.12.004 26763759

[B106] Merz R. A. Woodin S. A. (2006). Polychaete chaetae: function, fossils, and phylogeny. Integr. Comp. Biol. 46, 481–496. 10.1093/icb/icj057 21672760

[B107] Meysman F. J. Middelburg J. J. Heip C. H. (2006). Bioturbation: a fresh look at Darwin’s last idea. Trends Ecol. Evol. 21, 688–695. 10.1016/j.tree.2006.08.002 16901581

[B108] Montana J. Finn J. K. Norman M. D. (2015). Liquid sand burrowing and mucus utilisation as novel adaptations to a structurally-simple environment in octopus kaurna stranks, 1990. Behaviour 152, 1871–1881. 10.1163/1568539X-00003313

[B109] Murphy E. A. K. Dorgan K. M. (2011). Burrow extension with a proboscis: mechanics of burrowing by the glycerid Hemipodus simplex. J. Exp. Biol. 214, 1017–1027. 10.1242/jeb.051227 21346130

[B110] Murphy R. R. (2004). Activities of the rescue robots at the world trade center from 11-21 september 2001. IEEE Robotics and Automation Mag. 11, 50–61. 10.1109/MRA.2004.1337826

[B111] Naclerio N. D. Karsai A. Murray-Cooper M. Ozkan-Aydin Y. Aydin E. Goldman D. I. (2021). Controlling subterranean forces enables a fast, steerable, burrowing soft robot. Sci. Robotics 6, eabe2922. 10.1126/scirobotics.abe2922 34135117

[B112] Nagai M. Hirabayashi C. Yamada Y. Nakamura T. Yoshida H. (2017). Development of a flexible excavation unit for a peristaltic crawling seabed excavation robot. World Sci., 97–105. 10.1142/9789813149137_0015

[B113] Nel R. McLachlan A. Winter D. P. (2001). The effect of grain size on the burrowing of two Donax species. J. Exp. Mar. Biol. Ecol. 265, 219–238. 10.1016/S0022-0981(01)00335-5

[B114] Niiyama R. Matsushita K. Ikeda M. Or K. Kuniyoshi Y. (2022). A 3d printed hydrostatic skeleton for an earthworm-inspired soft burrowing robot. Soft Matter 18, 7990–7997. 10.1039/d2sm00882c 36218365

[B115] Olaf K. Marco S. Fittock M. Georgios T. Torben W. Lars W. (2019). Design details of the hp3 mole onboard the insight mission. Acta Astronaut. 164, 152–167. 10.1016/j.actaastro.2019.06.031

[B116] Omori H. Nakamura T. Yada T. (2009). An underground explorer robot based on peristaltic crawling of earthworms. Industrial Robot An Int. J. 36, 358–364. 10.1108/01439910910957129

[B117] Ortiz D. Gravish N. Tolley M. T. (2019). Soft robot actuation strategies for locomotion in granular substrates. IEEE Robotics Automation Lett. 4, 2630–2636. 10.1109/LRA.2019.2911844

[B118] Ozkan-Aydin Y. Liu B. Ferrero A. C. Seidel M. Hammond F. L. Goldman D. I. (2021a). Lateral bending and buckling aids biological and robotic earthworm anchoring and locomotion. Bioinspiration and Biomimetics 17, 016001. 10.1088/1748-3190/AC24BF 34496355

[B119] Ozkan-Aydin Y. Liu B. Ferrero A. C. Seidel M. Hammond III F. L. Goldman D. I. (2021b). Lateral undulation aids biological and robotic earthworm anchoring and locomotion. bioRxiv 2021. 10.1101/2021.02.02.429151 34496355

[B120] Ozkan-Aydin Y. Murray-Cooper M. Aydin E. McCaskey E. N. Naclerio N. Hawkes E. W. (2019). “Nutation aids heterogeneous substrate exploration in a robophysical root,” in 2019 2nd IEEE international conference on soft robotics (RoboSoft) (IEEE), 172–177.

[B121] Palikaras K. Tavernarakis N. (2013). “ *Caenorhabditis elegans* (nematode),” in Brenner’s encyclopedia of genetics. Editors Maloy S. Hughes K. Second Edition) (San Diego: Academic Press), 404–408. 10.1016/B978-0-12-374984-0.00186-8

[B122] Pettibone M. H. (1963). Marine polychaete worms of the new england region. i. aphroditidae through trochochaetidae. Bull. U. S. Natl. Mus., 1–356. 10.5479/si.03629236.227.1

[B123] Pierce C. J. Irvine D. Peng L. Lu X. Lu H. Goldman D. I. (2024). Dispersion relations for active undulators in overdamped environments. ArXiv. 10.48550/arXiv.2407.13037 42493985

[B124] Qi X. Shi H. Pinto T. Tan X. (2020). A novel pneumatic soft snake robot using traveling-wave locomotion in constrained environments. IEEE Robotics Automation Lett. 5, 1610–1617. 10.1109/lra.2020.2969923

[B125] Rieser J. M. Chong B. Gong C. Astley H. C. Schiebel P. E. Diaz K. (2024). Geometric phase predicts locomotion performance in undulating living systems across scales. Proc. Natl. Acad. Sci. 121, e2320517121. 10.1073/pnas.2320517121 38848301 PMC11181092

[B126] Rieser J. M. Li T.-D. Tingle J. L. Goldman D. I. Mendelson J. R. (2021). Functional consequences of convergently evolved microscopic skin features on snake locomotion. Proc. Natl. Acad. Sci. 118, e2018264118. 10.1073/pnas.2018264118 33547241 PMC8017952

[B127] Riisgard H. Banta G. T. (1998). Irrigation and deposit feeding by the lugworm arenicola marina, characteristics and secondary effects on the environment. a review of current knowledge. Vie et Milieu/Life and Environment, 243–257.

[B128] Rizzo A. E. Steiner T. M. Amaral A. C. Z. (2007). Glyceridae grube 1850 (annelida: polychaeta) from southern and southeastern Brazil, including a new species of glycera. Biota Neotropica 7, 41–59. 10.1590/s1676-06032007000300005

[B129] Robertson P. K. Campanella R. G. (1983). Interpretation of cone penetration tests. Part II: clay. Can. Geotechnical J. 20, 734–745. 10.1139/t83-079

[B130] Ruiz S. Or D. Schymanski S. J. (2015). Soil penetration by earthworms and plant roots-mechanical energetics of bioturbation of compacted soils. PloS one 10, e0128914. 10.1371/journal.pone.0128914 26087130 PMC4472233

[B131] Ruiz S. Schymanski S. J. Or D. (2017). Mechanics and energetics of soil penetration by earthworms and plant roots: higher rates cost more. Vadose Zone J. 16, 1–16. 10.2136/vzj2017.01.0021

[B132] Russell R. A. (2011a). Crabot: a biomimetic burrowing robot designed for underground chemical source location. Adv. Robot. 25, 119–134. 10.1163/016918610X538516

[B133] Russell R. A. (2011b). CRABOT: a biomimetic burrowing robot designed for underground chemical source location. Adv. Robot. 25, 119–134. 10.1163/016918610X538516

[B134] Sadeghi A. Mondini A. Mazzolai B. (2017). Toward self-growing soft robots inspired by plant roots and based on additive manufacturing technologies. Soft Robot. 4, 211–223. 10.1089/soro.2016.0080 29062628 PMC5649421

[B135] Sadeghi A. Tonazzini A. Popova L. Mazzolai B. (2013). “Robotic mechanism for soil penetration inspired by plant root,” in Proceedings - IEEE international conference on robotics and automation, 3457–3462. 10.1109/ICRA.2013.6631060

[B136] Seymour M. K. (1971). Burrowing behaviour in the european lugworm arenicola marina (polychaeta: arenicolidae). J. Zoology 164, 93–132. 10.1111/j.1469-7998.1971.tb01299.x

[B137] Sharpe S. S. Koehler S. A. Kuckuk R. M. Serrano M. Vela P. A. Mendelson I. (2015). Locomotor benefits of being a slender and slick sand swimmer. J. Exp. Biol. 218, 440–450. 10.1242/jeb.108357 25524983

[B138] Shepherd R. F. Stokes A. A. Nunes R. M. D. Whitesides G. M. (2013). Soft machines that are resistant to puncture and that self seal. Adv. Mater 25, 6709–6713. 10.1002/adma.201303175 24123311

[B139] SpaceX (2021). Falcon user’s guide

[B140] Stanley S. M. (1969). Bivalve mollusk burrowing aided by discordant shell ornamentation. Science 166, 634–635. 10.1126/science.166.3905.634 17778205

[B141] Steendam C. Verhelst P. Van Wassenbergh S. De Meyer J. (2020). Burrowing behaviour of the European eel (*Anguilla anguilla*): effects of life stage. J. Fish Biol. 97, 1332–1342. 10.1111/jfb.14481 32740934

[B142] Su H. Hou X. Zhang X. Qi W. Cai S. Xiong X. (2022). Pneumatic soft robots: challenges and benefits. Actuators 11 11, 92. 10.3390/act11030092

[B143] Sun L. Li J. Chen Y. Yang Y. Tao Y. Wang G. (2020). 4dtexture: a shape-changing fabrication method for 3d surfaces with texture. Ext. Abstr. 2020 CHI Conf. Hum. Factors Comput. Syst., 1–7. 10.1145/3334480.3383053

[B144] Suter R. B. Stratton G. E. Miller P. R. (2011). Mechanics and energetics of excavation by burrowing wolf spiders, geolycosa spp. J. Insect Sci. 11, 1–15. 10.1673/031.011.0122 21529154 PMC3281395

[B145] Tang Y. Zhong Y. Tao J. (2024). Bio-inspired rotational penetration and horizontal self-burrowing soft robot. Acta Geotech. 19, 1345–1363. 10.1007/s11440-023-02173-z

[B146] Tao J. â. Huang S. Tang Y. (2019). Bioinspired self-burrowing-out robot in dry sand. J. Geotechnical Geoenvironmental Eng. 145, 02819002. 10.1061/(asce)gt.1943-5606.0002177

[B147] Tao J. J. Huang S. Tang Y. (2020). SBOR: a minimalistic soft self-burrowing-out robot inspired by razor clams. Bioinspiration Biomimetics 15, 055003. 10.1088/1748-3190/ab8754 32259805

[B148] Tatom-Naecker T. A. M. Westneat M. W. (2018). Burrowing fishes: kinematics, morphology and phylogeny of sand-diving wrasses (Labridae). J. Fish Biol. 93, 860–873. 10.1111/jfb.13789 30175499

[B149] Taylor I. Lehner K. McCaskey E. Nirmal N. Ozkan-Aydin Y. Murray-Cooper M. (2021). Mechanism and function of root circumnutation, Proc. Natl. Acad. Sci. U. S. A., 118, e2018940118, 10.1073/pnas.2018940118 33608460 PMC7923379

[B150] Terzaghi K. Peck R. B. Mesri G. (1996). Soil mechanics in engineering practice. 3rd Edition.

[B151] Treers L. K. McInroe B. Full R. J. Stuart H. S. (2022). Mole crab-inspired vertical self-burrowing. Front. Robotics AI 9, 999392. 10.3389/frobt.2022.999392 PMC9594858 36304793

[B152] Trueman E. R. (1966). Bivalve mollusks: fluid dynamics of burrowing. Science 152, 523–525. 10.1126/science.152.3721.523 17815081

[B153] Trueman E. R. (1967). The dynamics of burrowing in Ensis (Bibalvia). Proc. R. Soc. Lond. Ser. B, Biol. Sci. 166, 459–476. 10.1098/rspb.1967.0007 24796040

[B154] Trueman E. R. (1975). The locomotion of soft-bodied animals

[B155] Trueman E. R. Brand A. R. Davis P. (1966). The dynamics of burrowing of some common littoral bivalves. J. Exp. Biol. 44, 469–492. 10.1242/jeb.44.3.469

[B156] Valentine A. D. Busbee T. A. Boley J. W. Raney J. R. Chortos A. Kotikian A. (2017). Hybrid 3d printing of soft electronics. Adv. Mater. 29, 1703817. 10.1002/adma.201703817 28875572

[B157] Volkenborn N. Polerecky L. Wethey D. S. Woodin S. A. (2010). Oscillatory porewater bioadvection in marine sediments induced by hydraulic activities of arenicola marina. Limnol. Oceanogr. 55, 1231–1247. 10.4319/lo.2010.55.3.1231

[B158] Wang F. Chen Y. Li Y. Li Y. (2024). A drag force model of vertical penetration into a granular medium based on dem simulations and experiments. Appl. Sci. 14, 2336. 10.3390/app14062336

[B159] Wang J. Chortos A. (2024). Performance metrics for shape-morphing devices. Nat. Rev. Mater. 9, 738–751. 10.1038/s41578-024-00714-w

[B160] Wehner M. Truby R. L. Fitzgerald D. J. Mosadegh B. Whitesides G. M. Lewis J. A. (2016). An integrated design and fabrication strategy for entirely soft, autonomous robots. nature 536, 451–455. 10.1038/nature19100 27558065

[B161] Wei H. Zhang Y. Zhang T. Guan Y. Xu K. Ding X. (2021). Review on bioinspired planetary regolith-burrowing robots. Space Sci. Rev. 217, 87–39. 10.1007/S11214-021-00863-2

[B162] Wickramanayake S. Thiyagarajan K. Kodagoda S. (2022). “Deep learned ground penetrating radar subsurface features for robot localization,” in 2022 IEEE sensors, 1–4. 10.1109/SENSORS52175.2022.9967350

[B163] Winter A. G. Deits R. L. Dorsch D. S. (2013) “Critical timescales for burrowing in undersea substrates via localized fluidization, demonstrated by RoboClam: a robot inspired by Atlantic razor clams,” in *Proceedings of the ASME design engineering technical conference* 6 A. 10.1115/DETC2013-12798

[B164] Winter A. G. Deits R. L. Dorsch D. S. Slocum A. H. Hosoi A. E. (2014). Razor clam to RoboClam: burrowing drag reduction mechanisms and their robotic adaptation. Bioinspiration Biomimetics 9, 036009. 10.1088/1748-3182/9/3/036009 24713848

[B165] Winter A. G. Deits R. L. Hosoi A. E. (2012). Localized fluidization burrowing mechanics of *Ensis directus* . J. Exp. Biol. 215, 2072–2080. 10.1242/jeb.058172 22623195

[B166] Woodin S. A. (1974). Polychaete abundance patterns in a marine soft-sediment environment: the importance of biological interactions. Ecol. Monogr. 44, 171–187. 10.2307/1942310

[B167] Woodman S. J. Shah D. S. Landesberg M. Agrawala A. Kramer-Bottiglio R. (2024). Stretchable arduinos embedded in soft robots. Sci. Robotics 9, eadn6844. 10.1126/scirobotics.adn6844 39259780

[B168] Wu W. Yu S. Schreiber P. Dollmann A. Lutz C. Gomard G. (2020). Variation of the frictional anisotropy on ventral scales of snakes caused by nanoscale steps. Bioinspiration and Biomimetics 15, 056014. 10.1088/1748-3190/ab9e51 32554875

[B169] Yang S. Qiu P. Chen L. Shi X. (2021). Recent developments in flexible thermoelectric devices. Small Sci. 1, 2100005. 10.1002/smsc.202100005 PMC11936022 40213054

[B170] Ye X. Zhang C. (2023). Impact granular media for intruders with different geometries: force and rheology. Acta Mech. Sin. 39, 722198. 10.1007/s10409-022-22198-x

[B171] Young B. A. Morain M. (2003). Vertical burrowing in the saharan sand vipers (cerastes). Copeia 2003, 131–137. 10.1643/0045-8511(2003)003[0131:vbitss]2.0.co;2

[B172] Yuan W. Dong S. Adelson E. H. (2017). Gelsight: high-resolution robot tactile sensors for estimating geometry and force. Sensors 17, 2762. 10.3390/s17122762 29186053 PMC5751610

[B173] Zaidi A. A. Müller C. (2017). Vertical drag force acting on intruders of different shapes in granular media. In EPJ Web Conf., 140, 02011, 10.1051/epjconf/201714002011

[B174] Zarrouk D. Sharf I. Shoham M. (2012). Conditions for worm-robot locomotion in a flexible environment: theory and experiments. IEEE Trans. Biomed. Eng. 59, 1057–1067. 10.1109/tbme.2011.2182612 22231667

[B175] Zhang B. Fan Y. Yang P. Cao T. Liao H. (2019). Worm-like soft robot for complicated tubular environments. Soft Robot. 6, 399–413. 10.1089/soro.2018.0088 31180823

[B176] Zhang J. Niu X. Croxford A. J. Drinkwater B. W. (2022). Strategies for guided acoustic wave inspection using mobile robots. Proc. R. Soc. A Math. Phys. Eng. Sci. 478, 20210762. 10.1098/rspa.2021.0762 PMC8889470 35273453

[B177] Zhang T. Wei H. Zheng H. Liang Z. Yang H. Zhang Y. (2024a). Mole-inspired robot burrowing with forelimbs for planetary soil exploration. Adv. Intell. Syst. 2300392doi. 10.1002/aisy.202300392

[B178] Zhang W. Huang R. Xiang J. Zhang N. (2024b). Recent advances in bio-inspired geotechnics: from burrowing strategy to underground structures. Gondwana Res. 130, 1–17. 10.1016/j.gr.2023.12.018

[B179] Zhang Y. Yang D. Yan P. Zhou P. Zou J. Gu G. (2021). Inchworm inspired multimodal soft robots with crawling, climbing, and transitioning locomotion. IEEE Trans. Robotics 38, 1806–1819. 10.1109/tro.2021.3115257

[B180] Zwarts L. Wanink J. S. (1989). Siphon size and burying depth in deposit- and suspension-feeding benthic bivalves. Mar. Biol. 100, 227–240. 10.1007/BF00391963

